# Supplementary material for: Irreversible inhibitors of the 3C protease of Coxsackie virus through templated assembly of protein-binding fragments
Source: Nat Commun. 2016 Sep 28;7:12761. doi: 10.1038/ncomms12761 (PMC5052702; doi:10.1038/ncomms12761)
Supplement: Supplementary Information — Supplementary Figures 1-36, Supplementary Tables 1-3, Supplementary Methods and Supplementary References. [file ncomms12761-s1.pdf]

Supplementary Figures

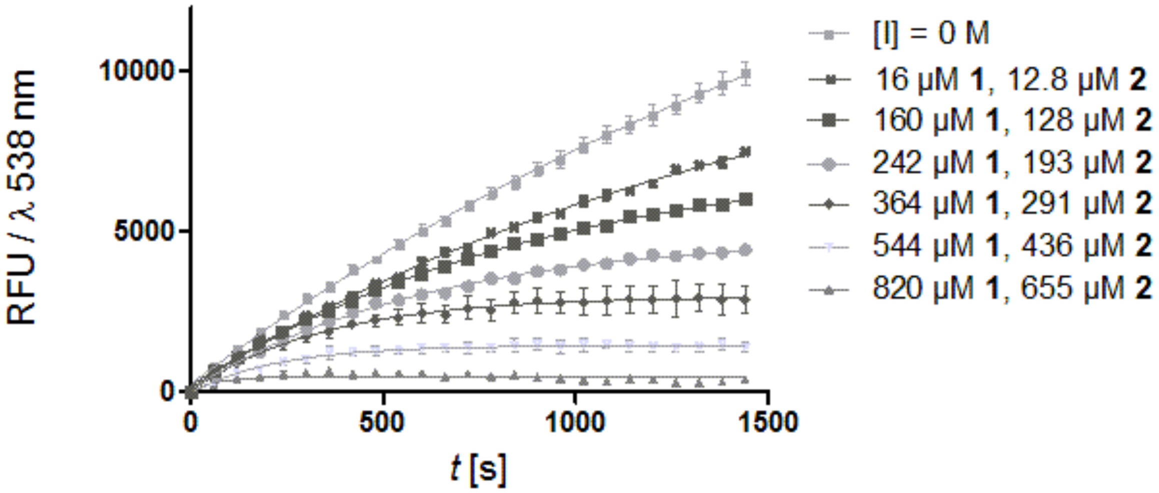

Supplementary Figure 1: Inhibition of the protease in the FRET-based enzyme activity assay at various concentrations of **1** and **2** in the same ratio as in the ligation assay**.**

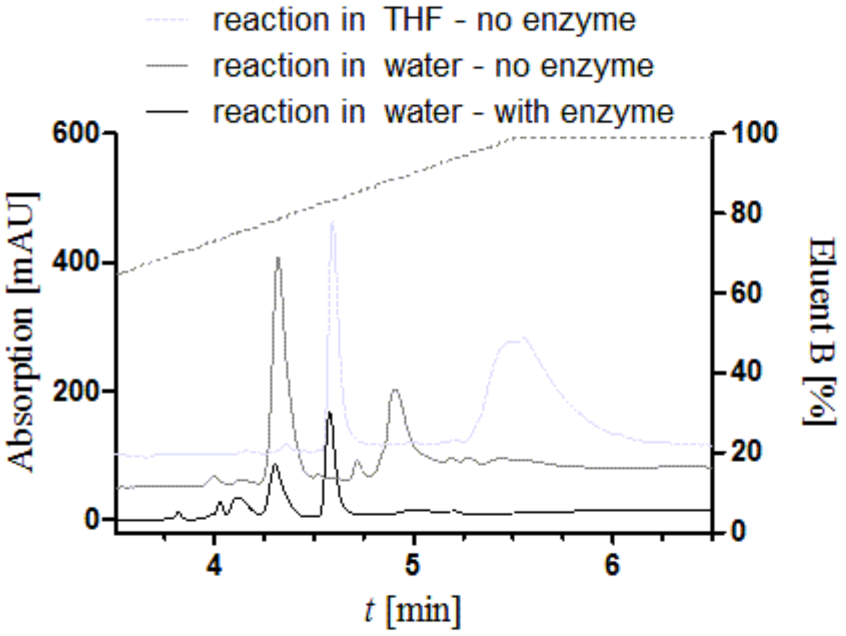

Supplementary Figure 2: The LC chromatogram of the ligation of **1** and **2** in different solvents and with or without enzyme present, clearly shows that multiple outcomes of the ligation process are possible and highly depend on the reaction conditions.

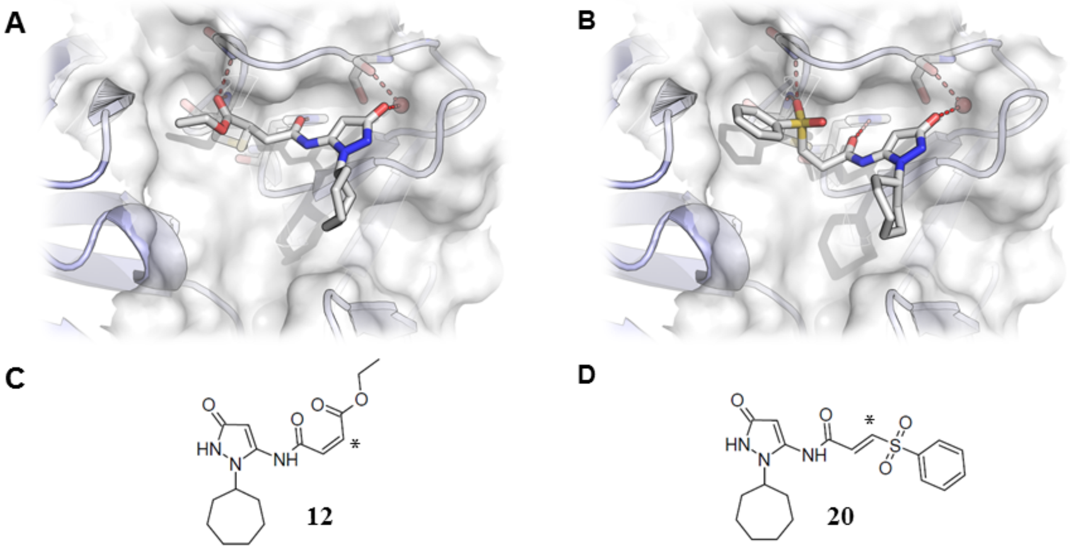

Supplementary Figure 3: A,B: Binding conformation of the inhibitors **12** (A) and **20** (B) in the active site of CVB3 3C protease generated by docking experiments. C,D: Structures of inhibitors **12** (C) and **20** (D) with the carbon participating in the covalent protease reaction highlighted by an asterisk.

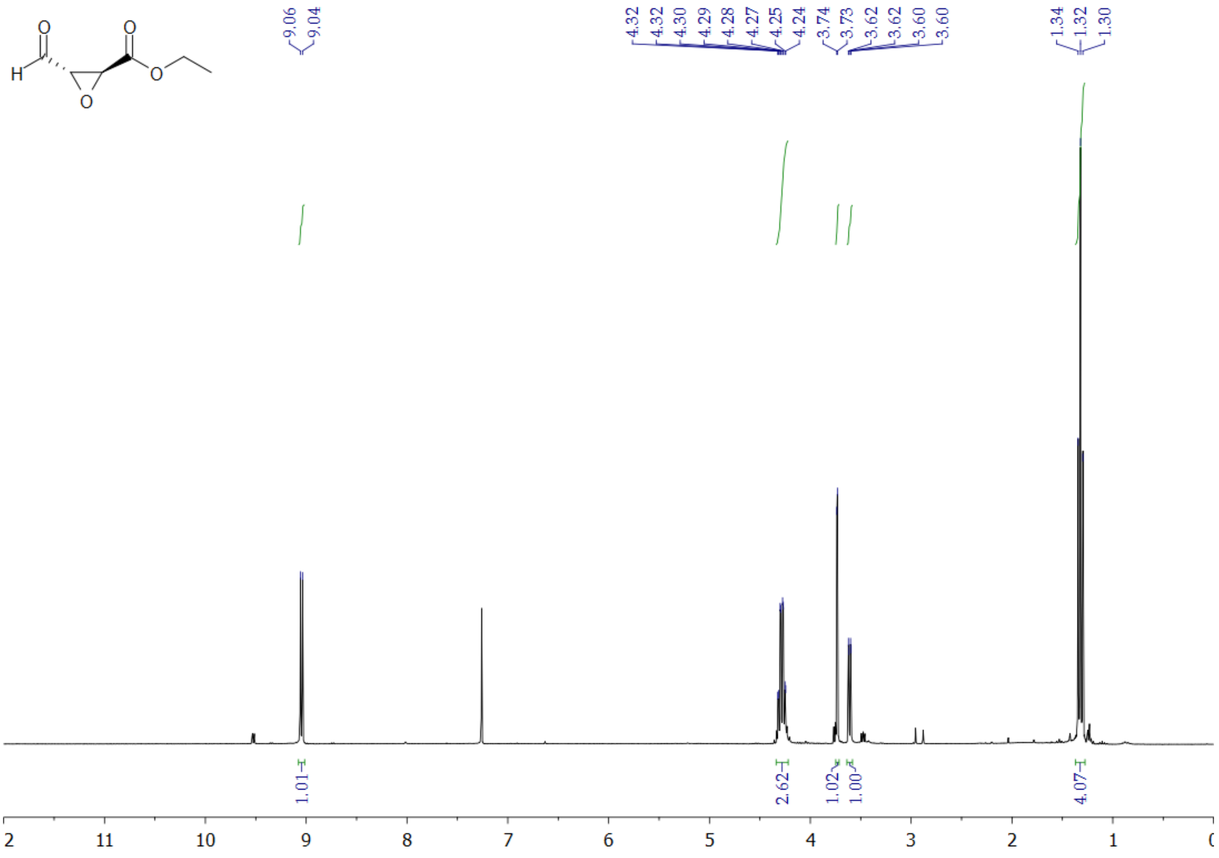

Supplementary Figure 4: ^{1}H-NMR spectrum of **1**

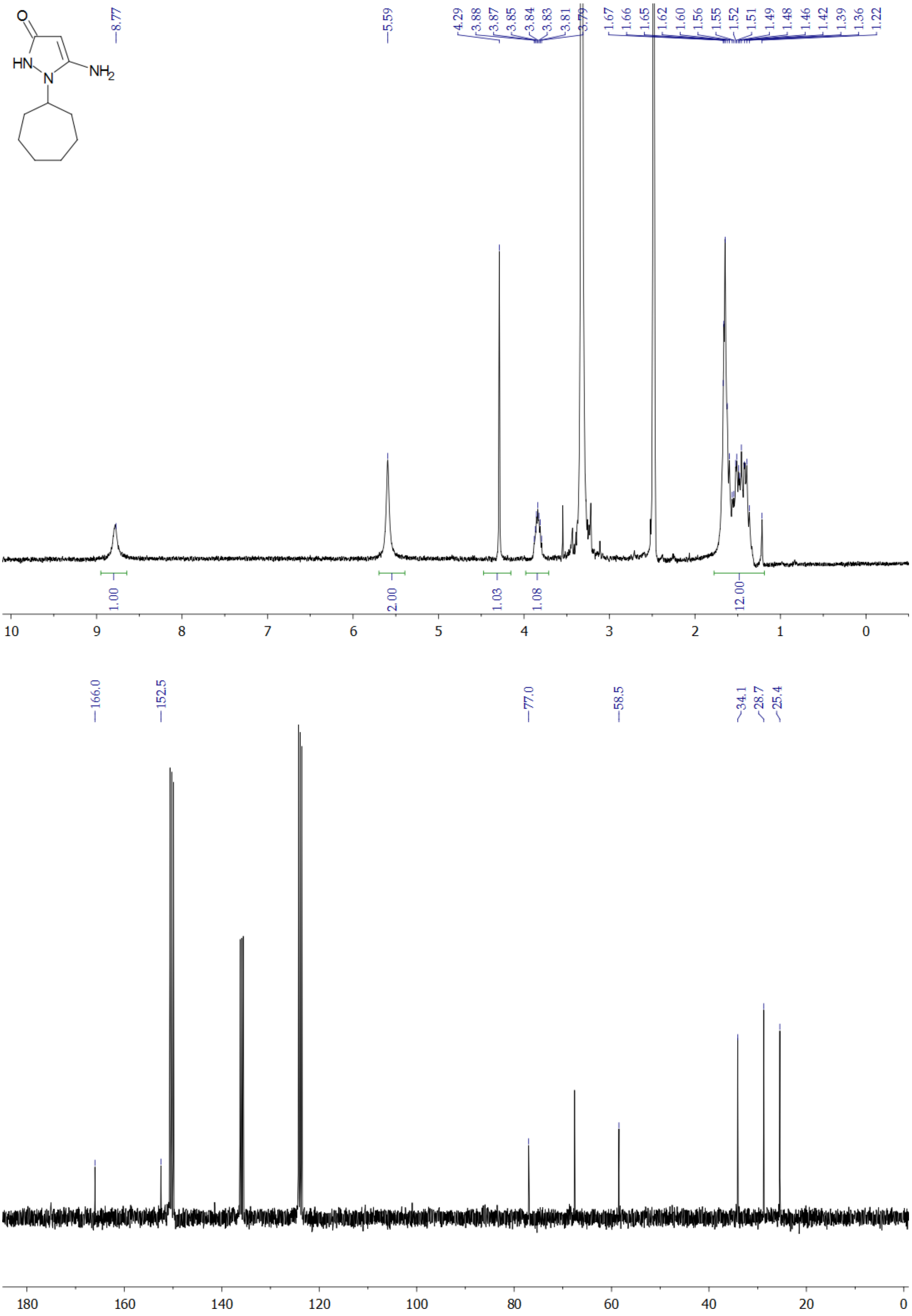

Supplementary Figure 5: ^{1}H- and ^{13}C-NMR spectrum of **2**

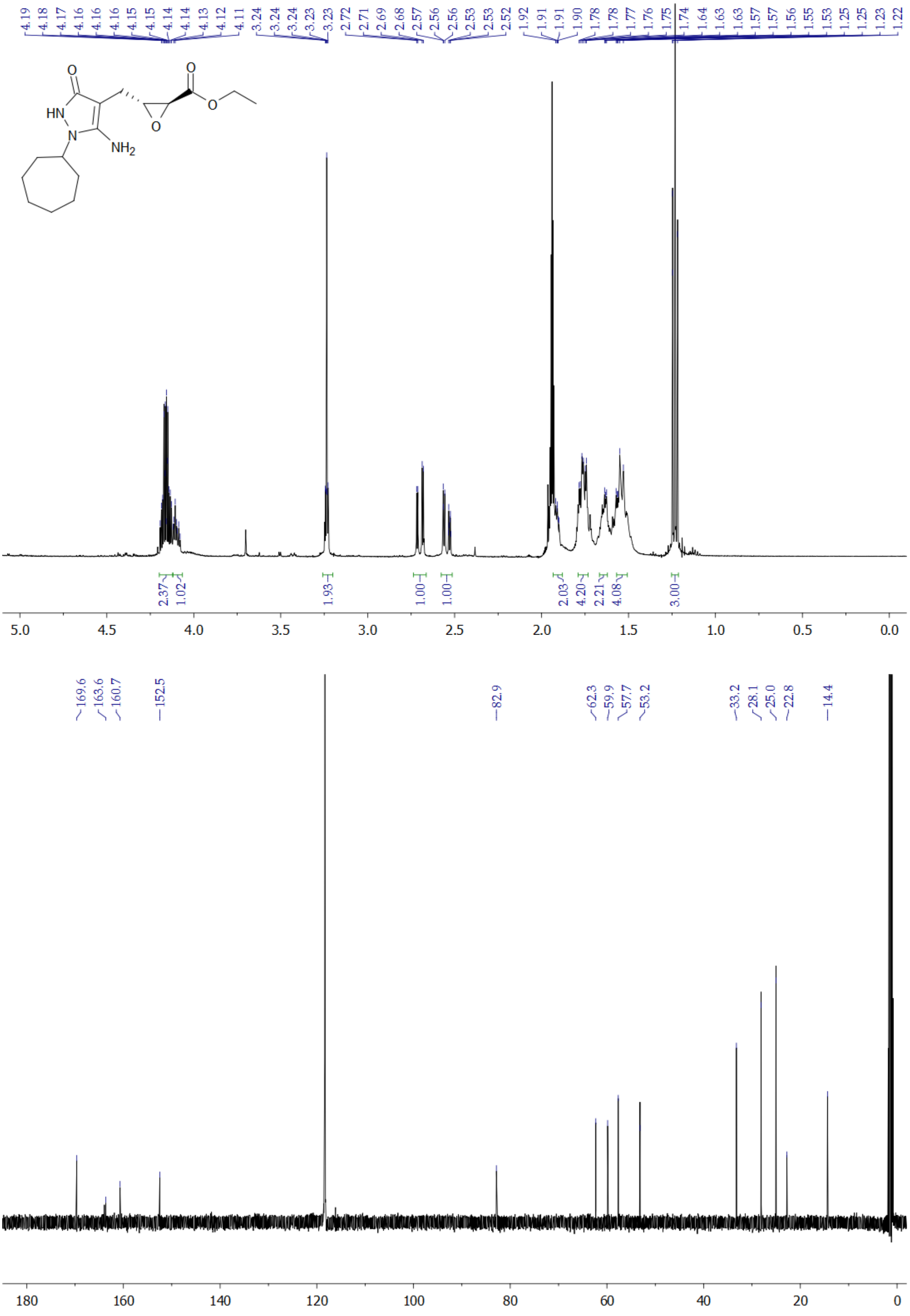

Supplementary Figure 6: ^{1}H- and ^{13}C-NMR spectrum of **3**

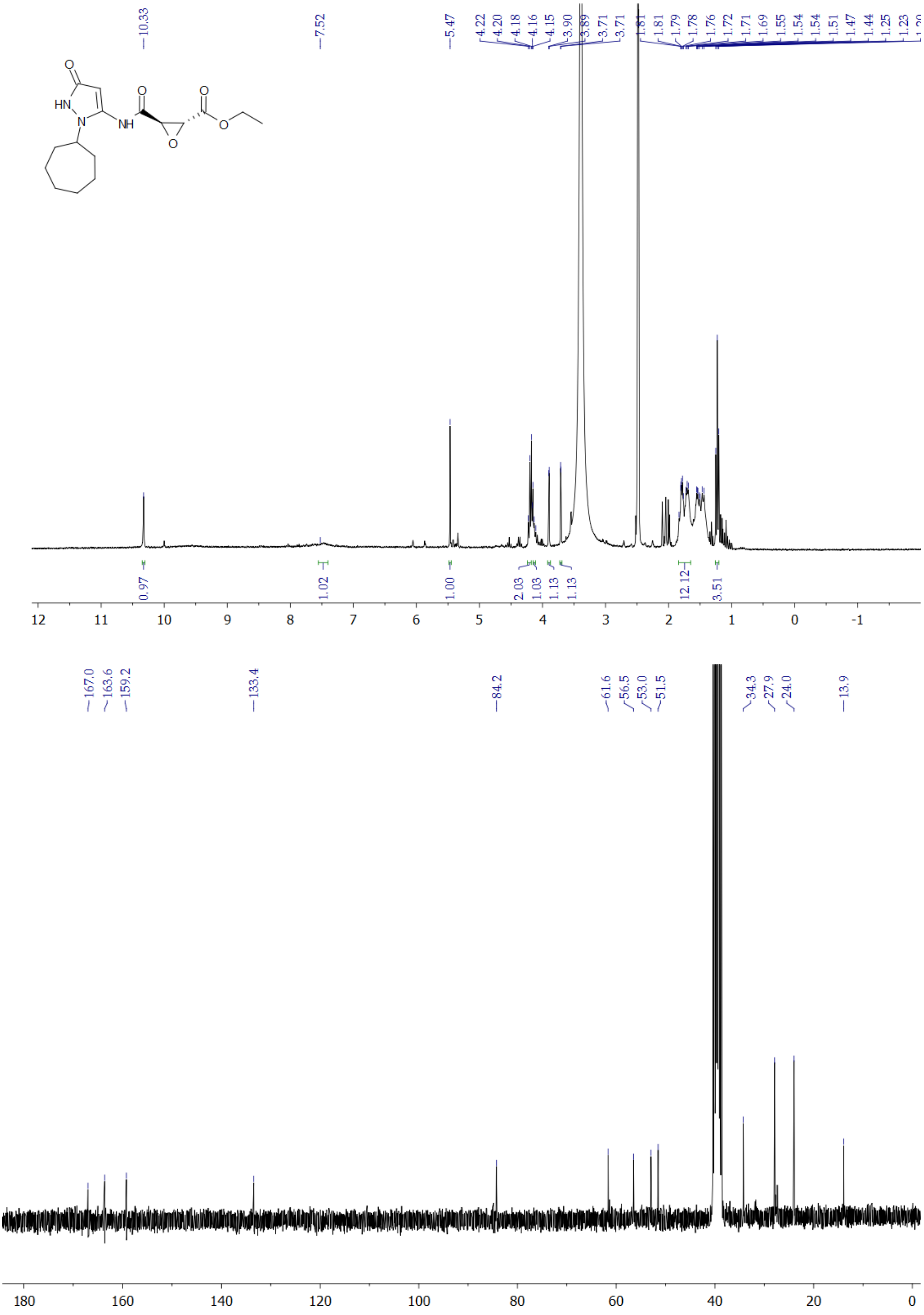

Supplementary Figure 7: ^{1}H- and ^{13}C-NMR spectrum of **4**

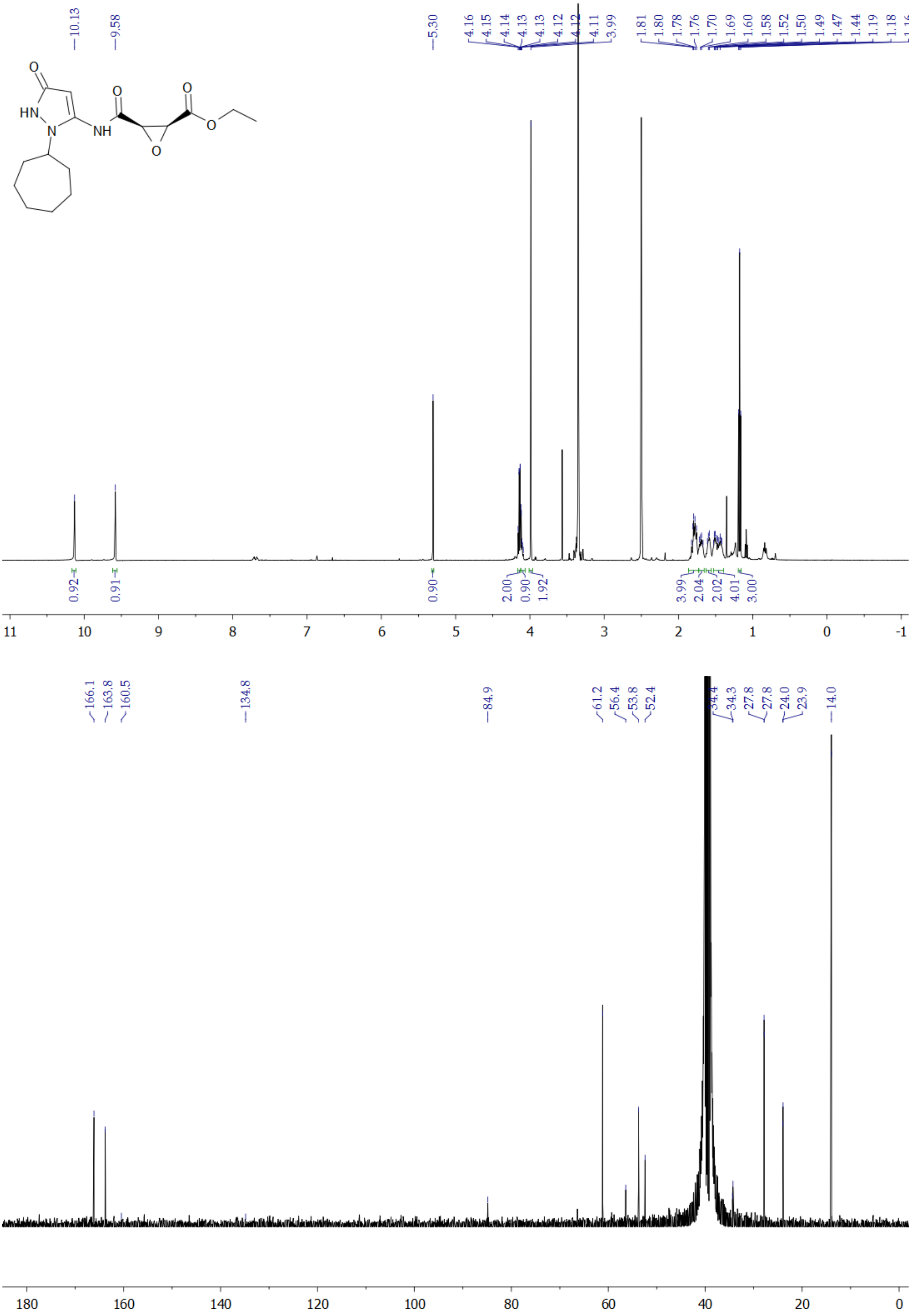

Supplementary Figure 8: ^{1}H- and ^{13}C-NMR spectrum of **5**

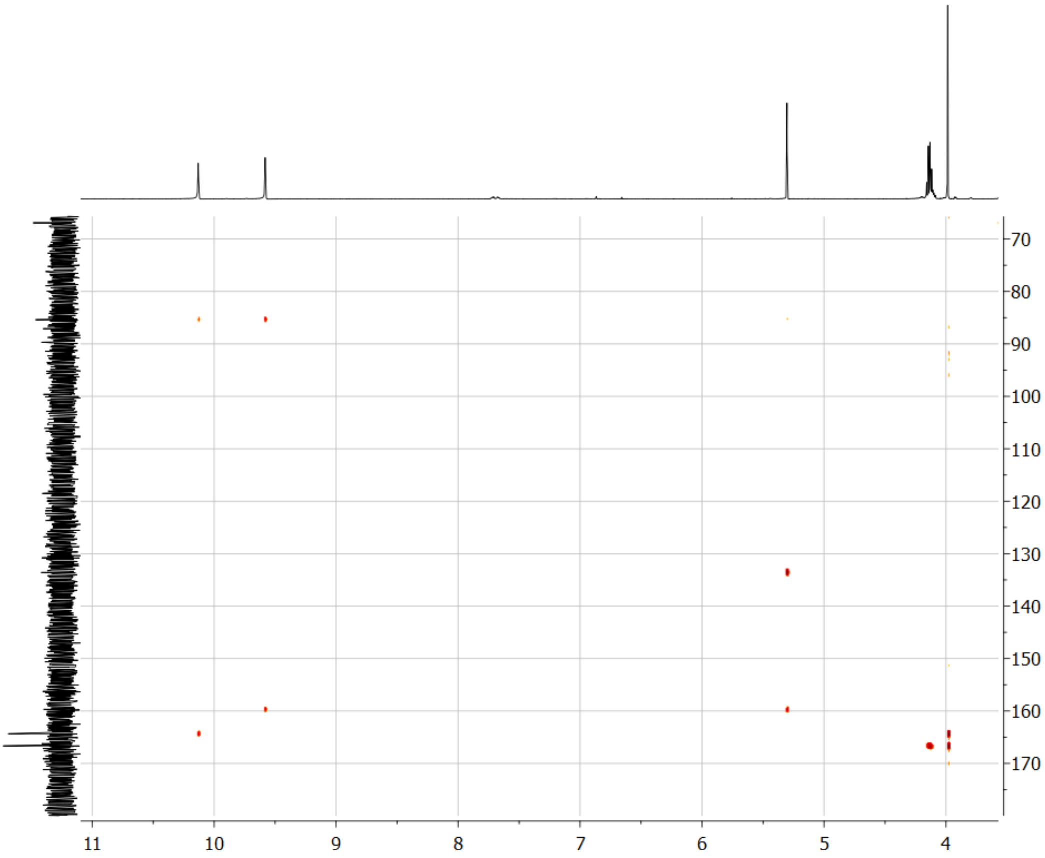

Supplementary Figure 9: [^{1}H,^{13}C]-HSQC-NMR spectrum of **5**

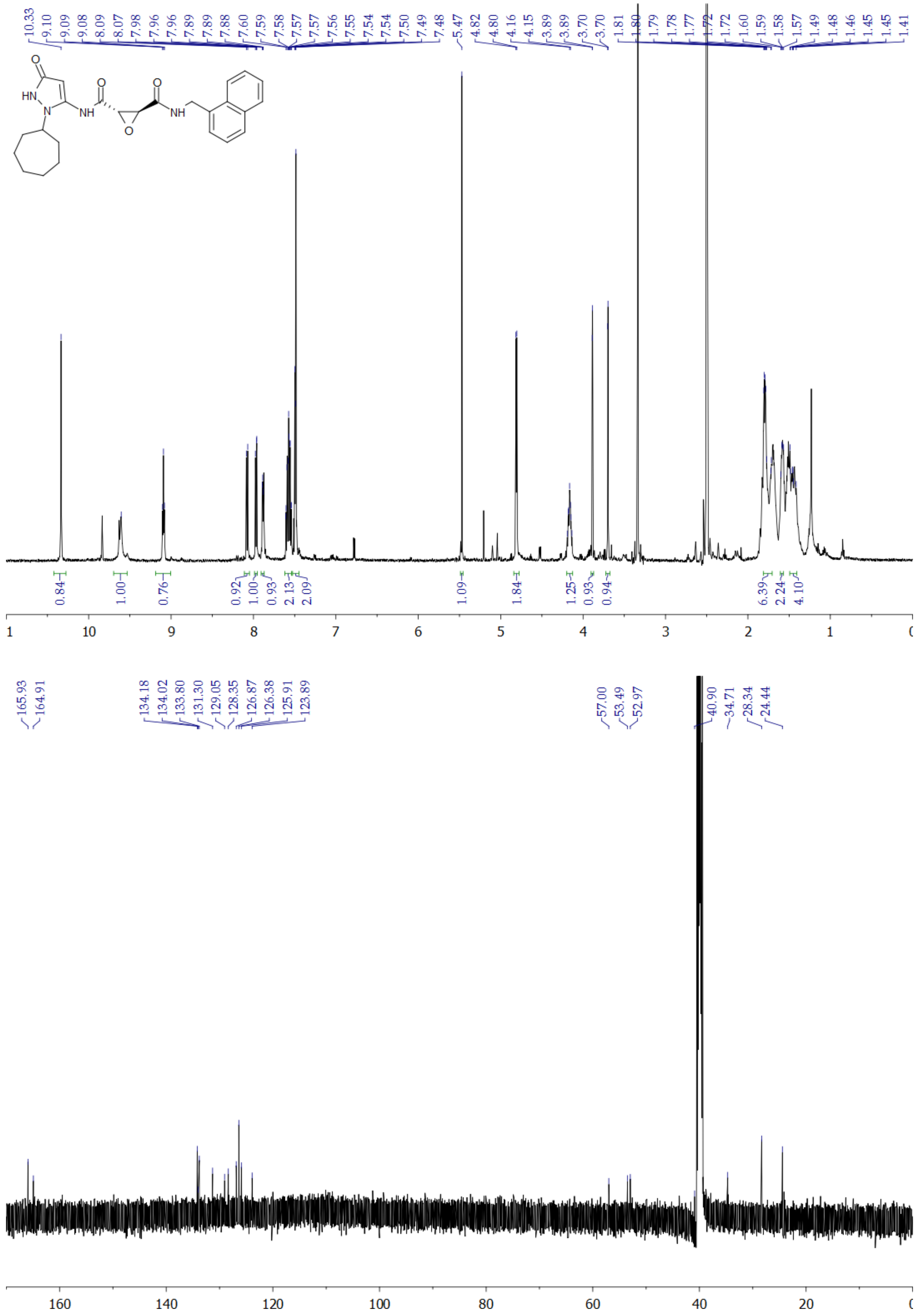

Supplementary Figure 10: ^{1}H- and ^{13}C-NMR spectrum of **6**

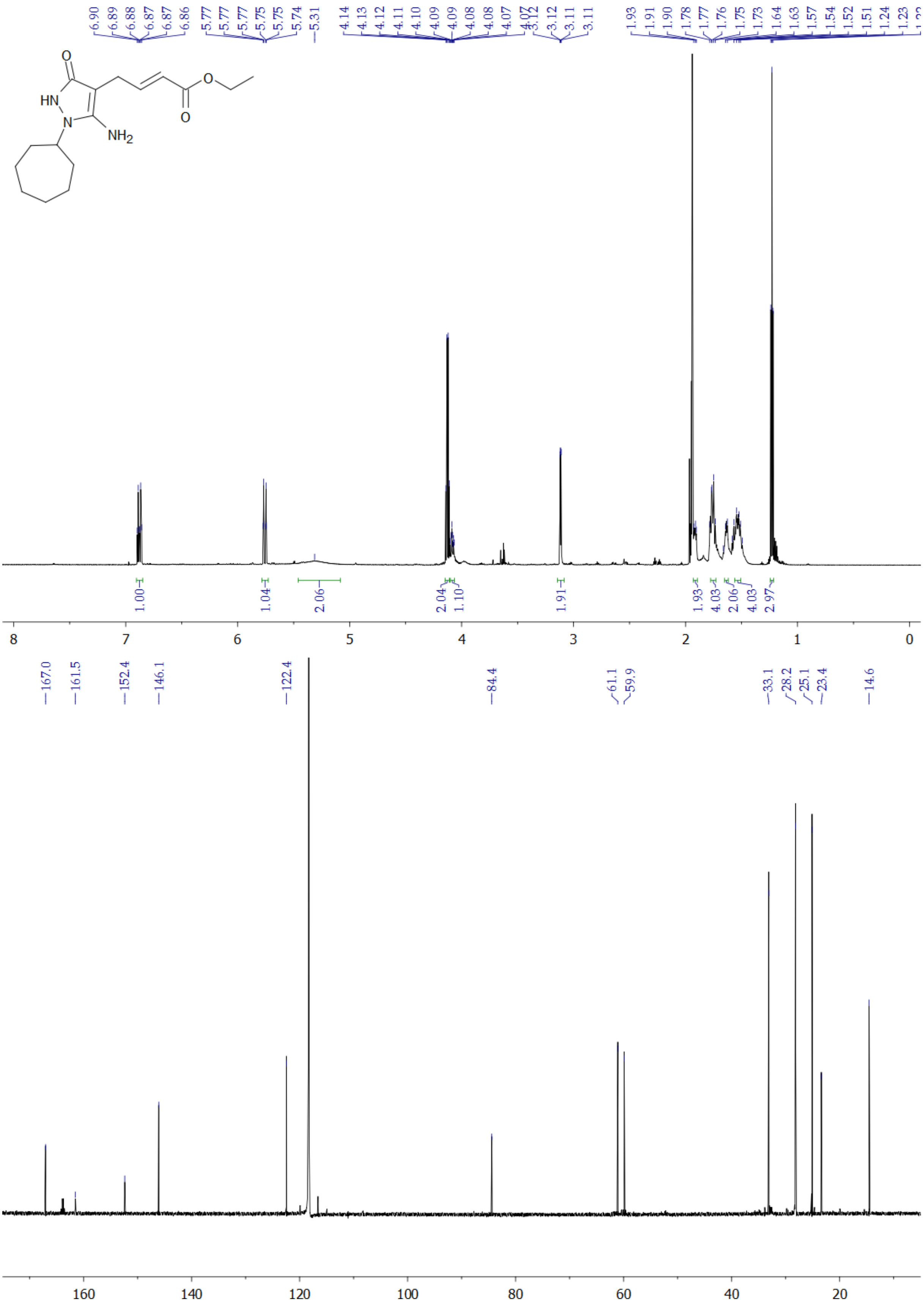

Supplementary Figure 11: ^{1}H- and ^{13}C-NMR spectrum of **7**

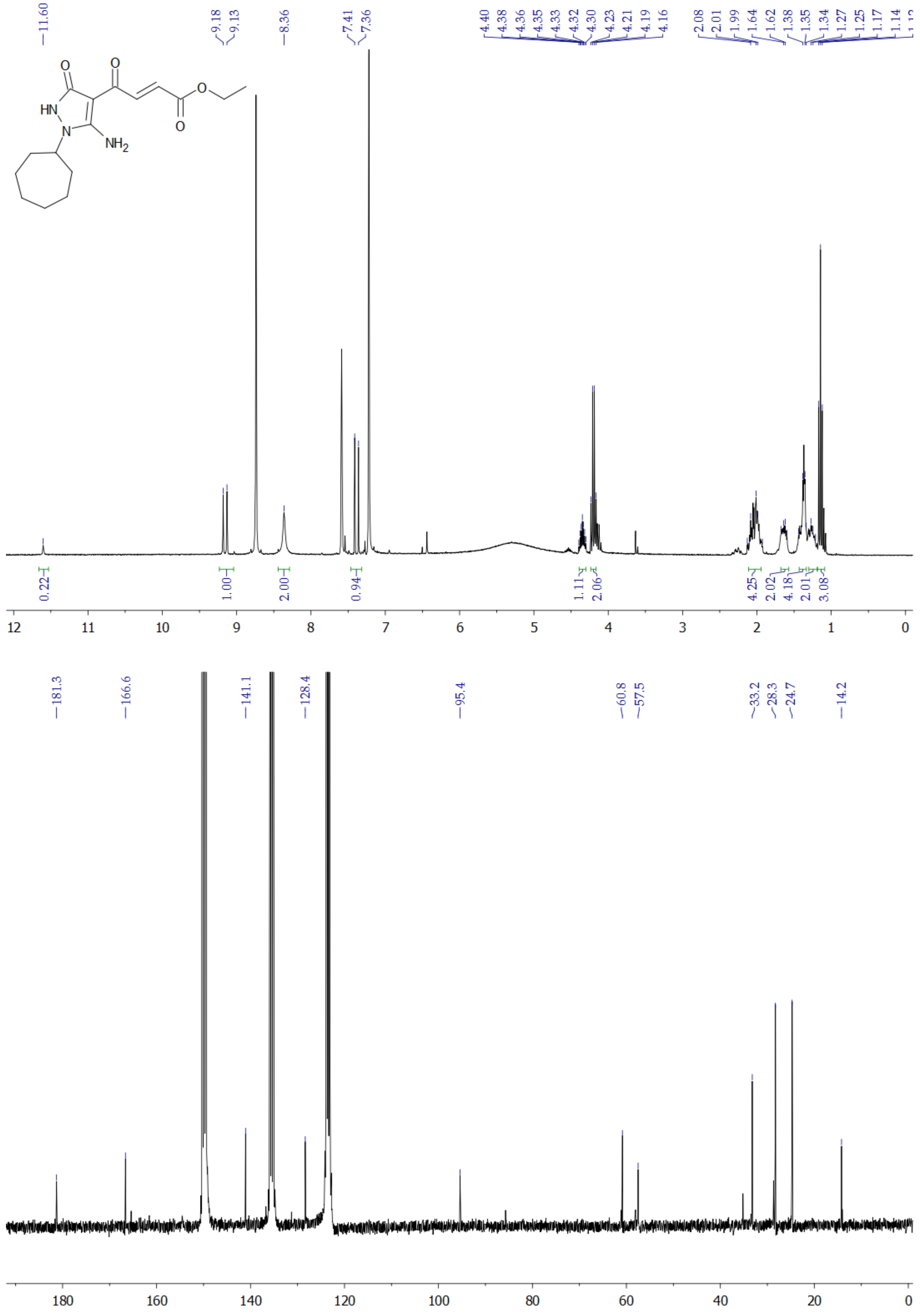

Supplementary Figure 12: ^{1}H- and ^{13}C-NMR spectrum of **8**

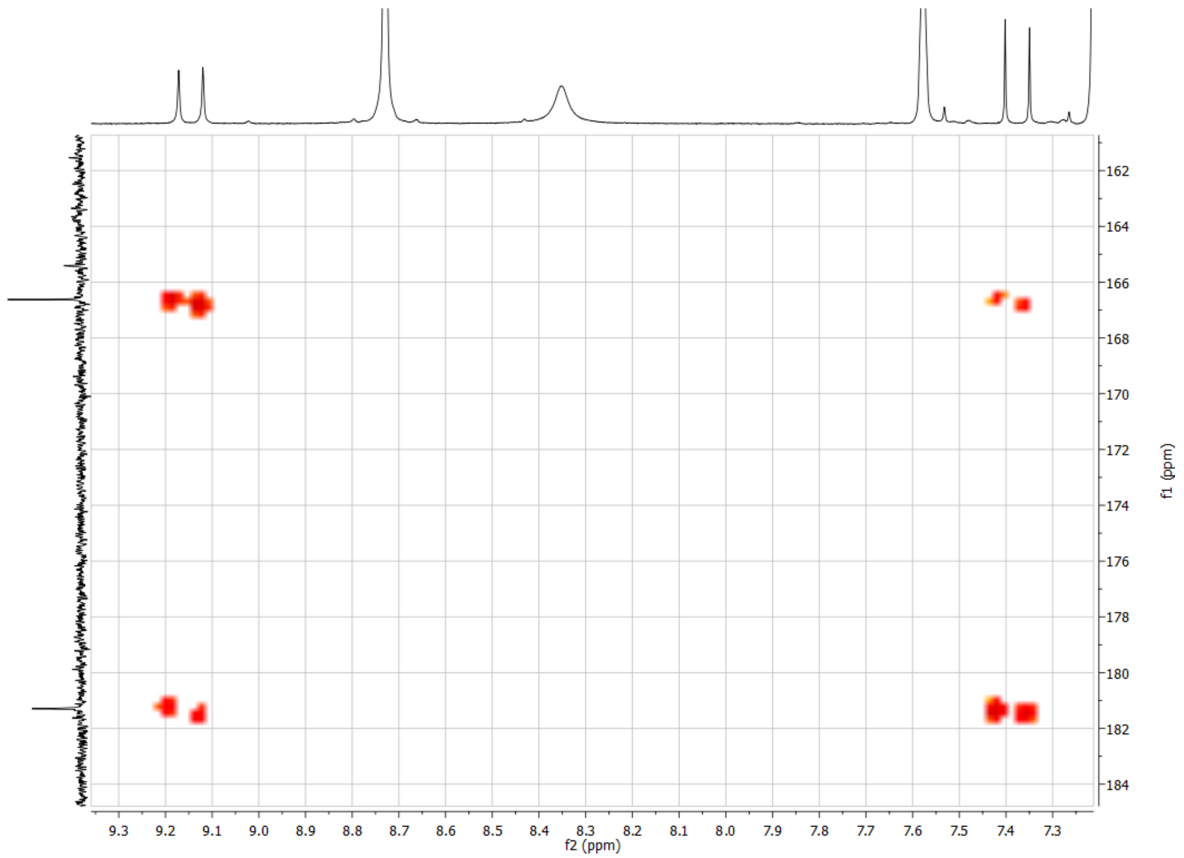

Supplementary Figure 13: [^{1}H,^{13}C]-HMBC-NMR spectrum of **8**

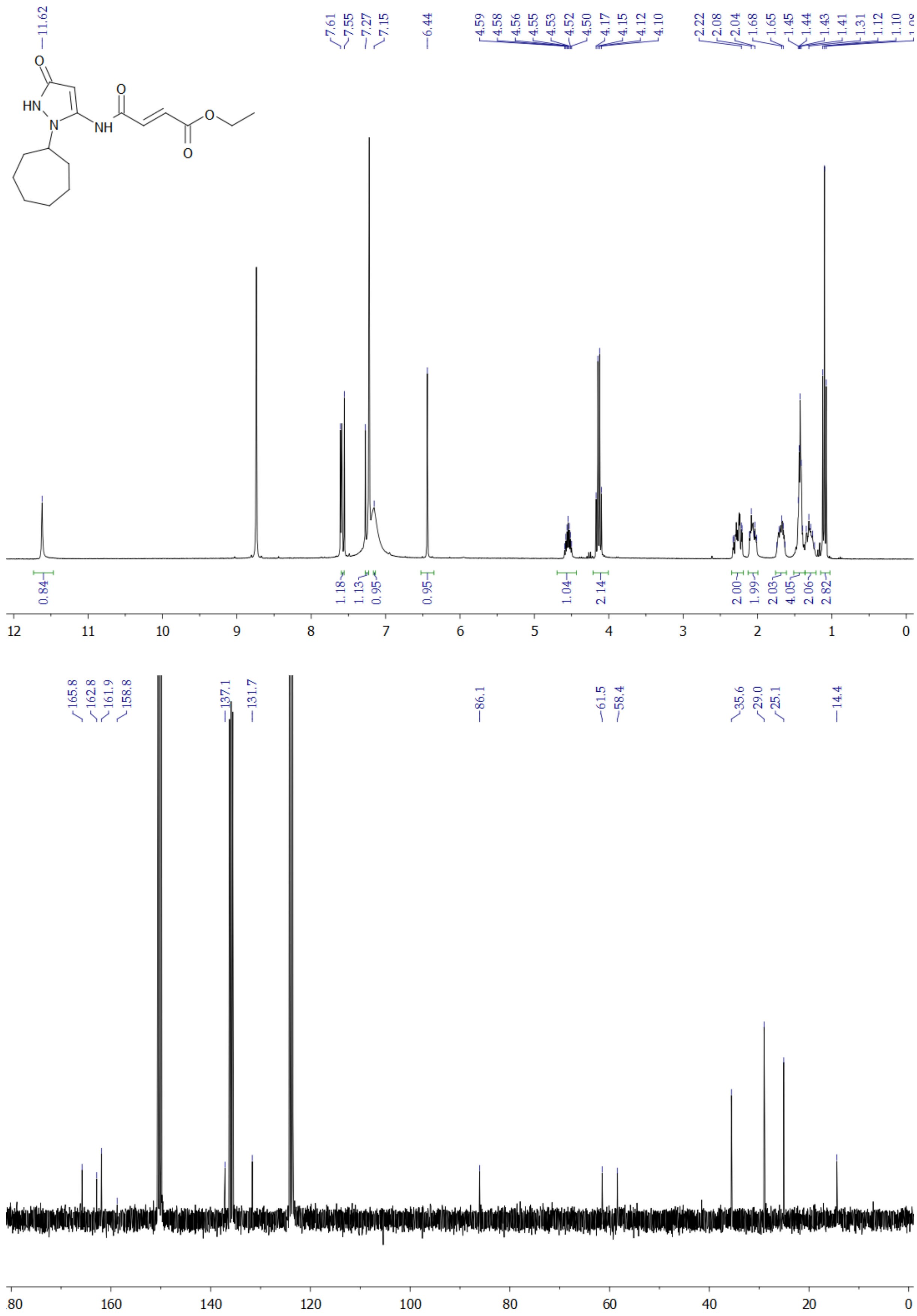

Supplementary Figure 14: ^{1}H- and ^{13}C-NMR spectrum of **9**

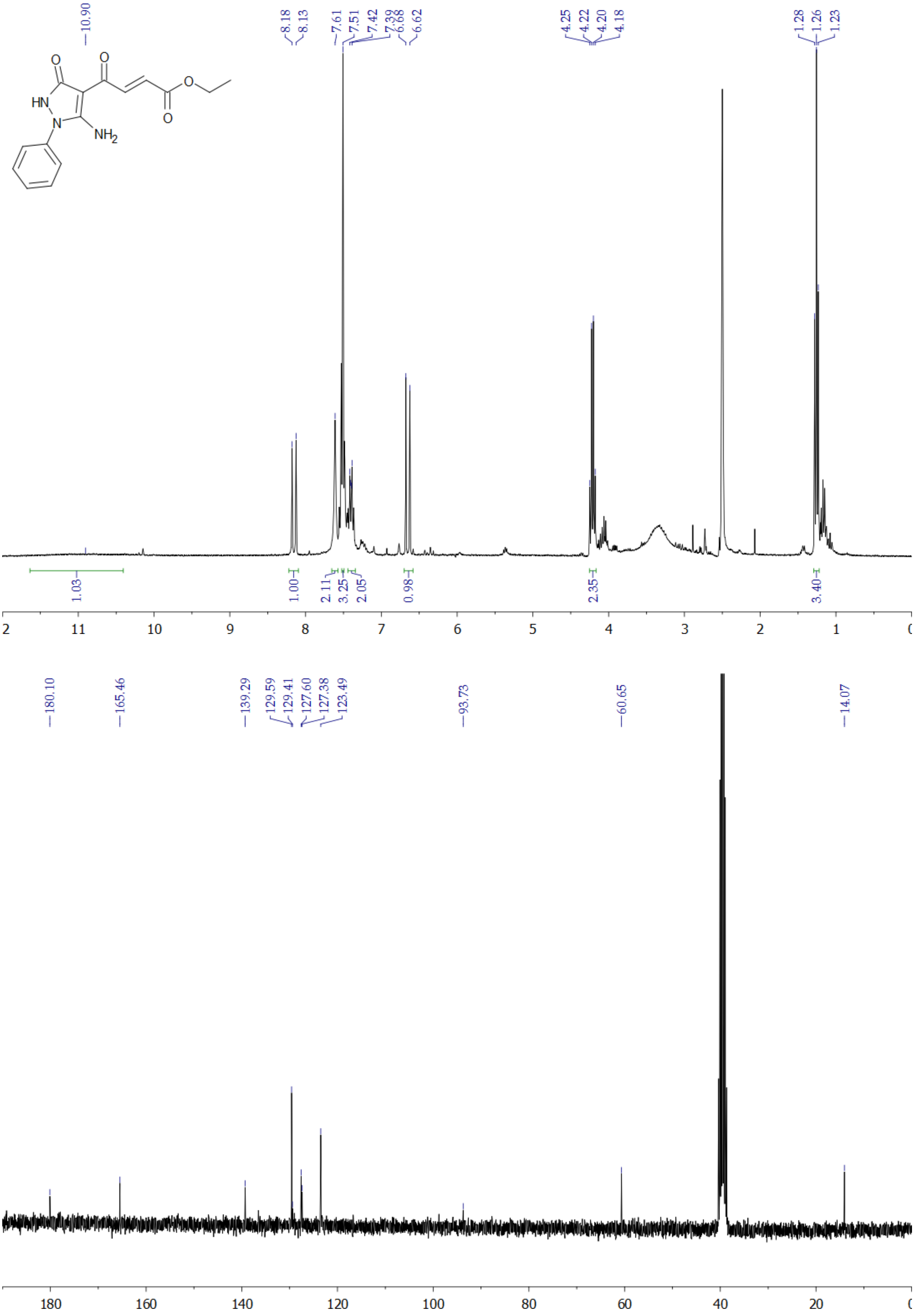

Supplementary Figure 15: ^{1}H- and ^{13}C-NMR spectrum of **10**

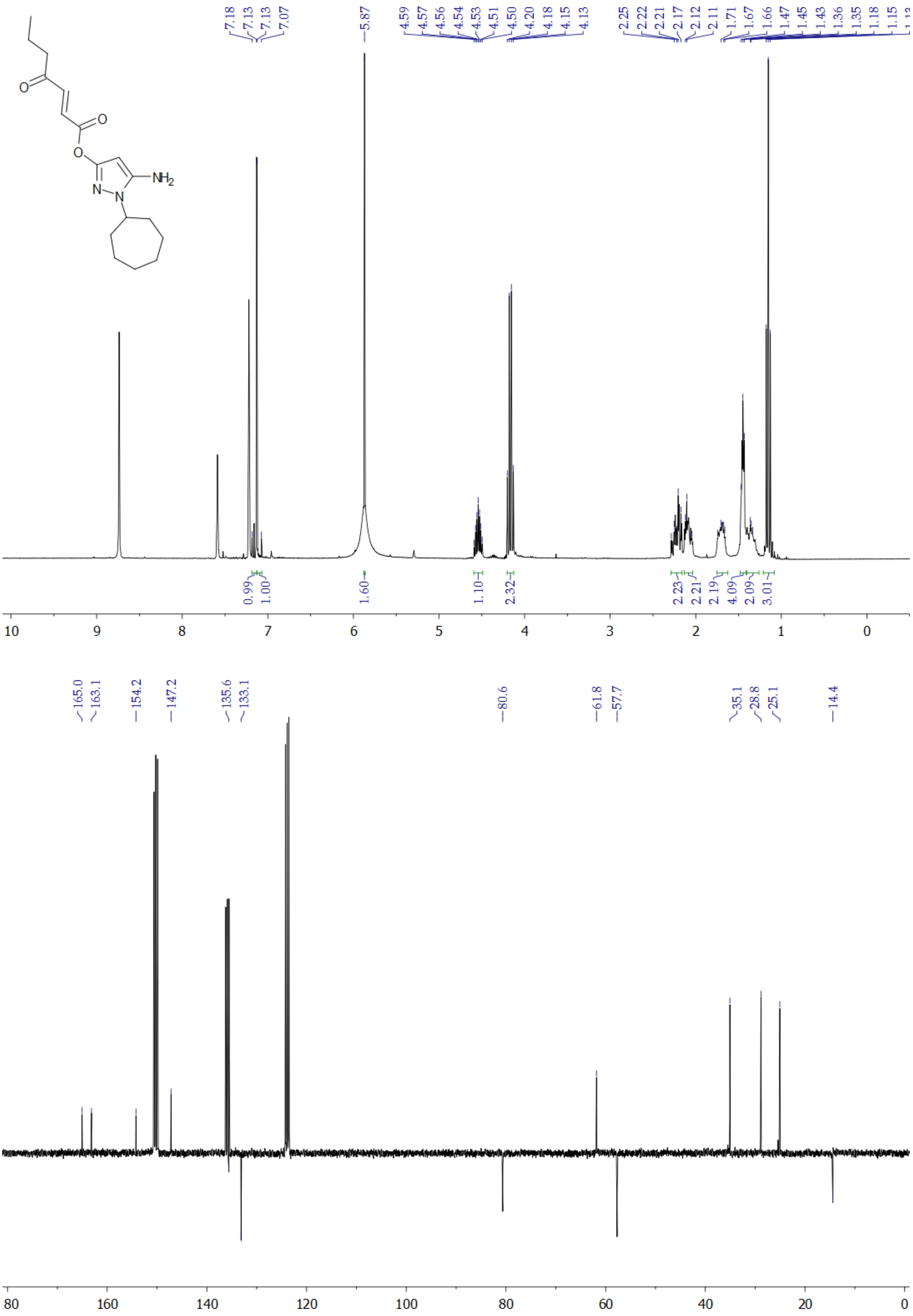

Supplementary Figure 16: ^{1}H- and ^{13}C-APT-NMR spectrum of **11**

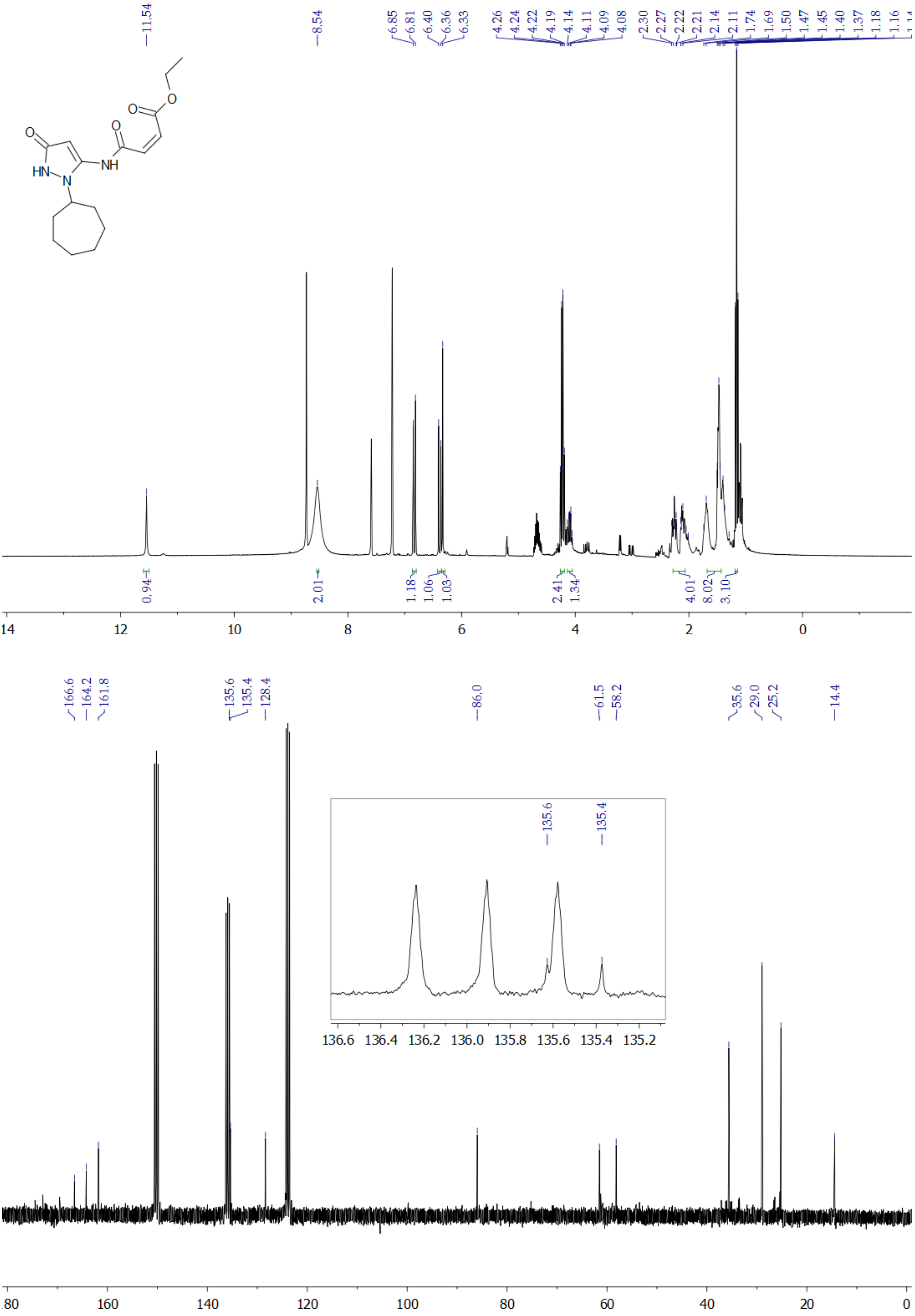

Supplementary Figure 17: ^{1}H- and ^{13}C-NMR spectrum of **12**

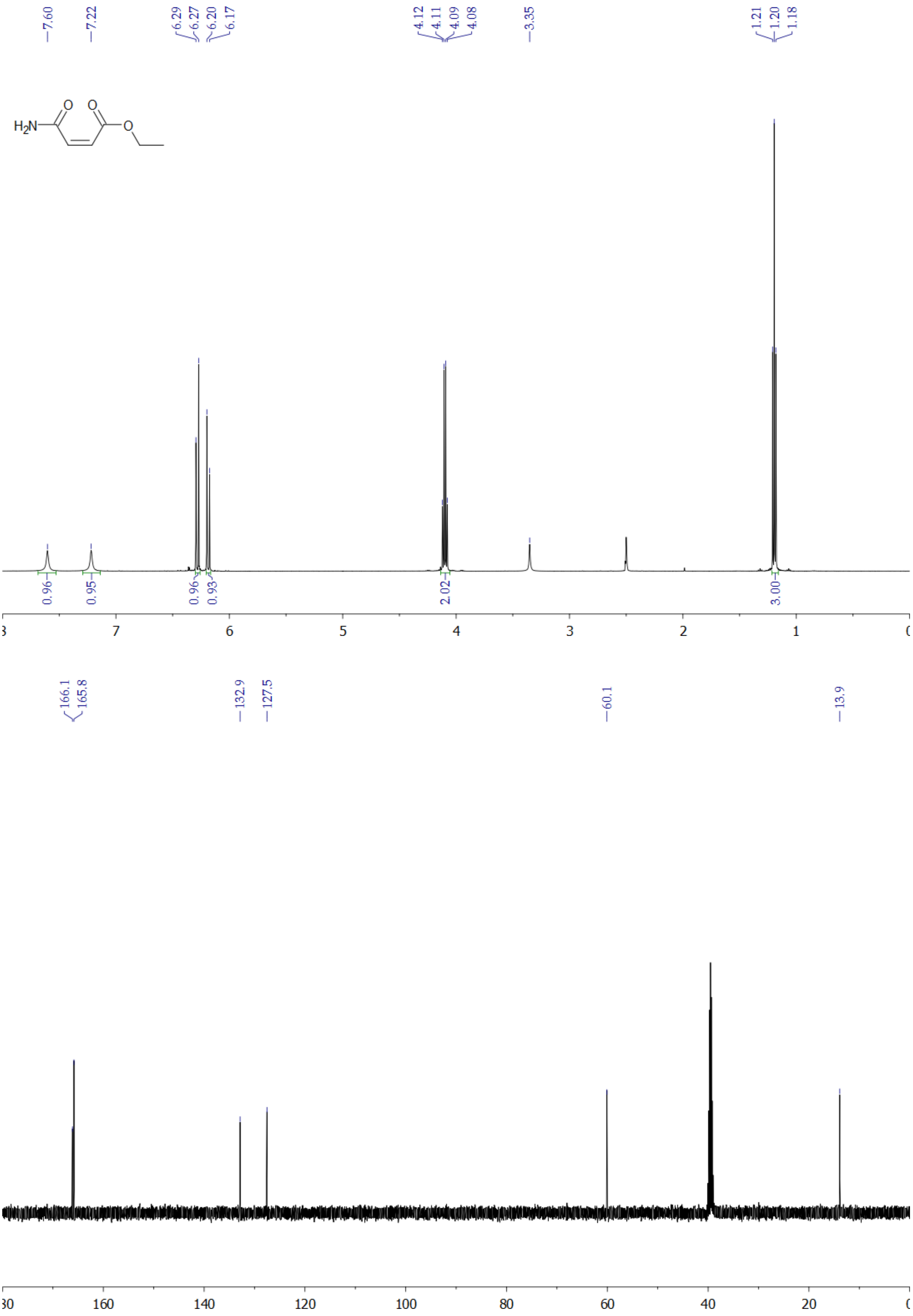

Supplementary Figure 18: ^{1}H- and ^{13}C-NMR spectrum of **13**

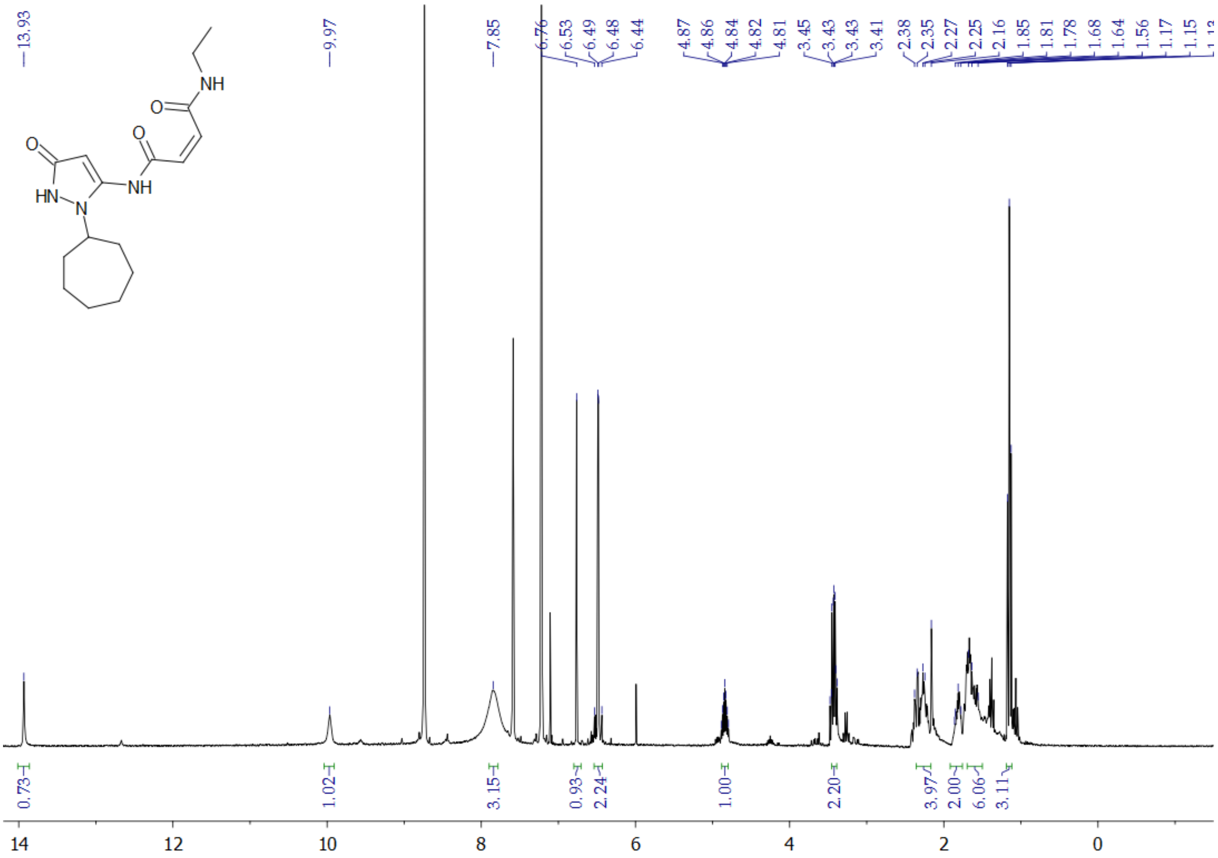

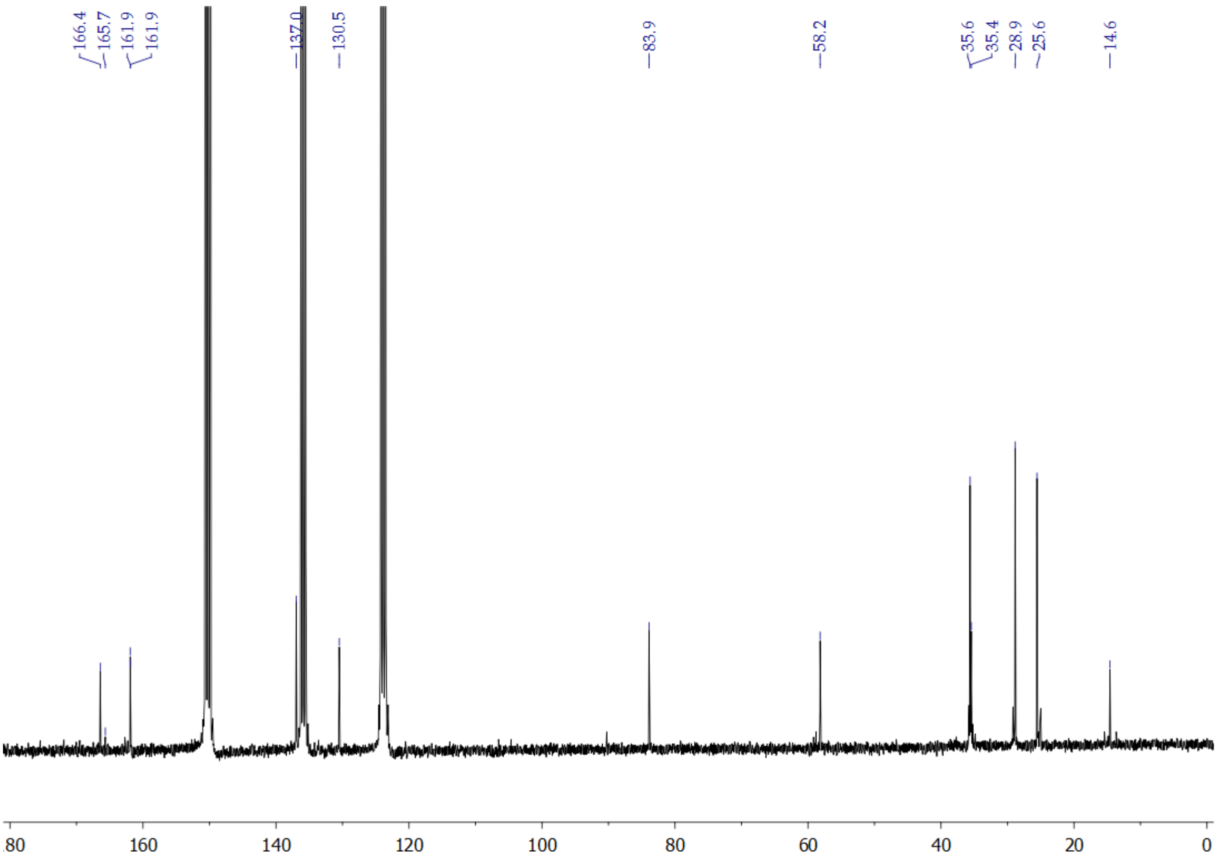

Supplementary Figure 19: ^{1}H- and ^{13}C-NMR spectrum of **14**

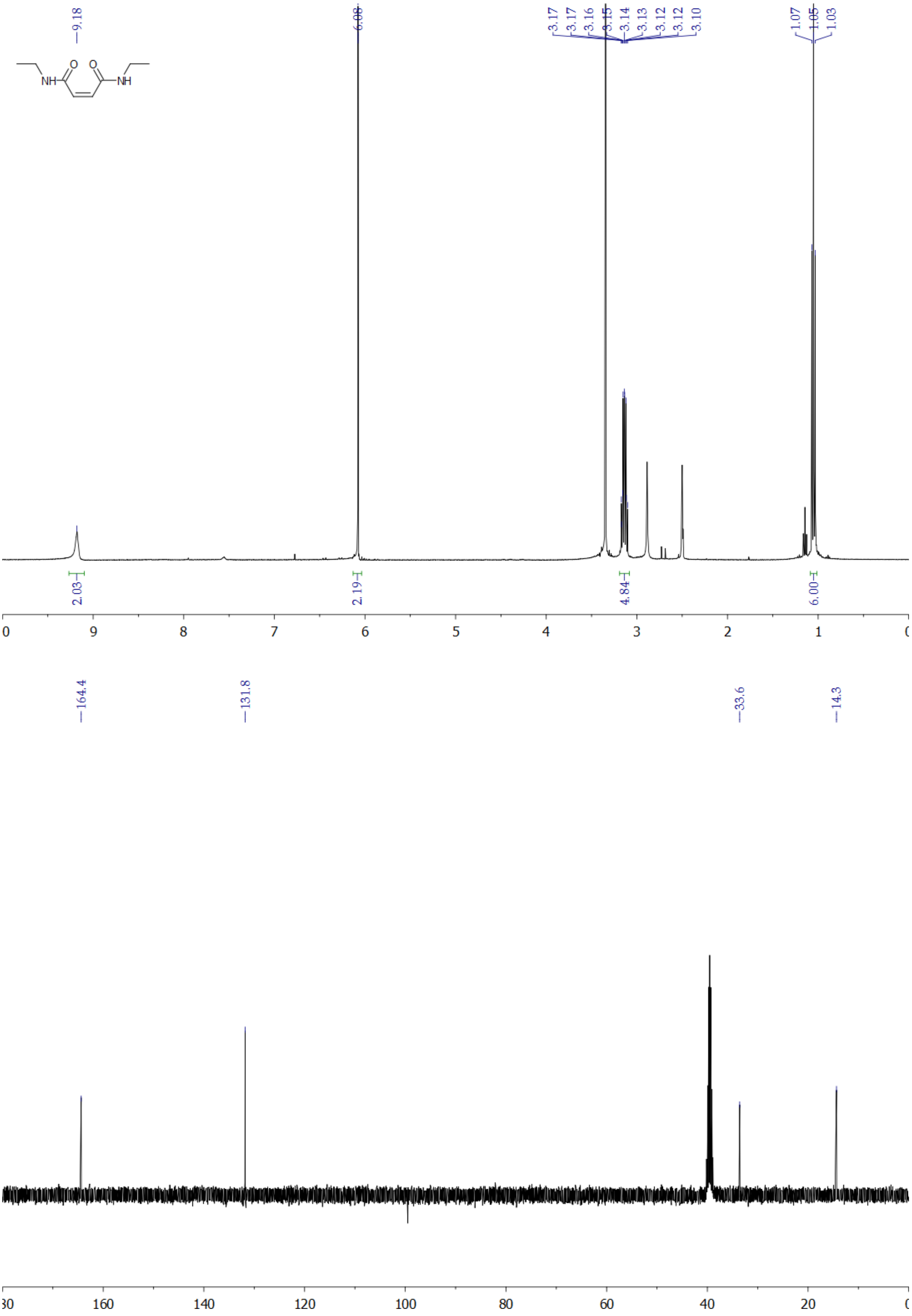

Supplementary Figure 20: ^{1}H- and ^{13}C-NMR spectrum of **15**

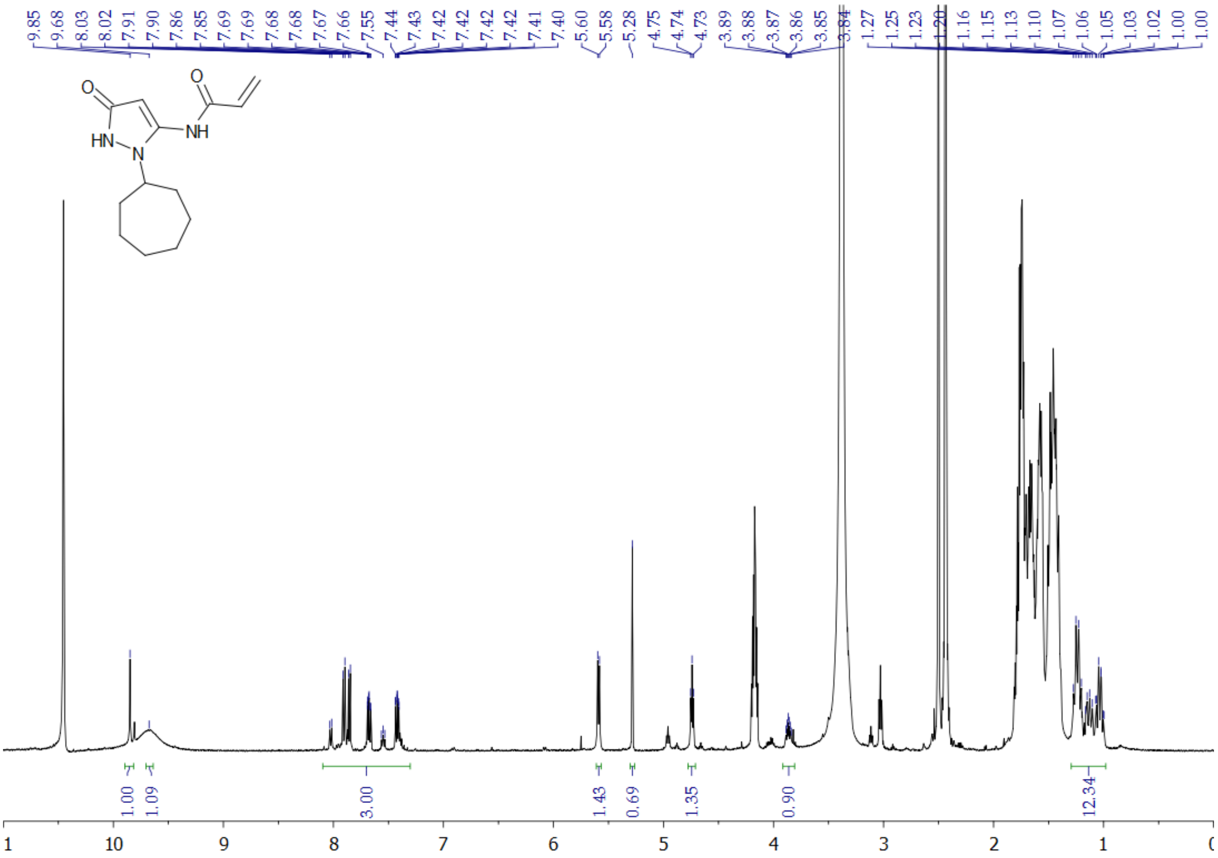

Supplementary Figure 21: ^{1}H-NMR spectrum of **16**

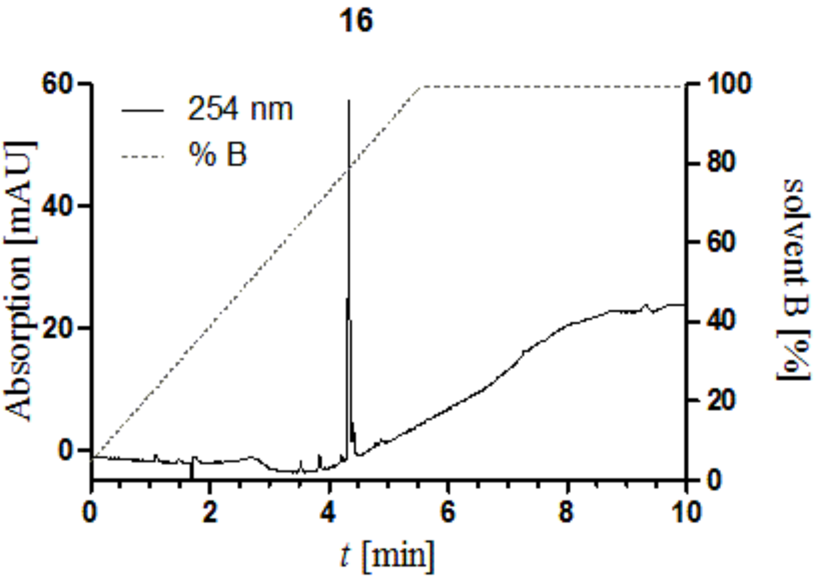

Supplementary Figure 22: RP-HPLC chromatogram of **16**

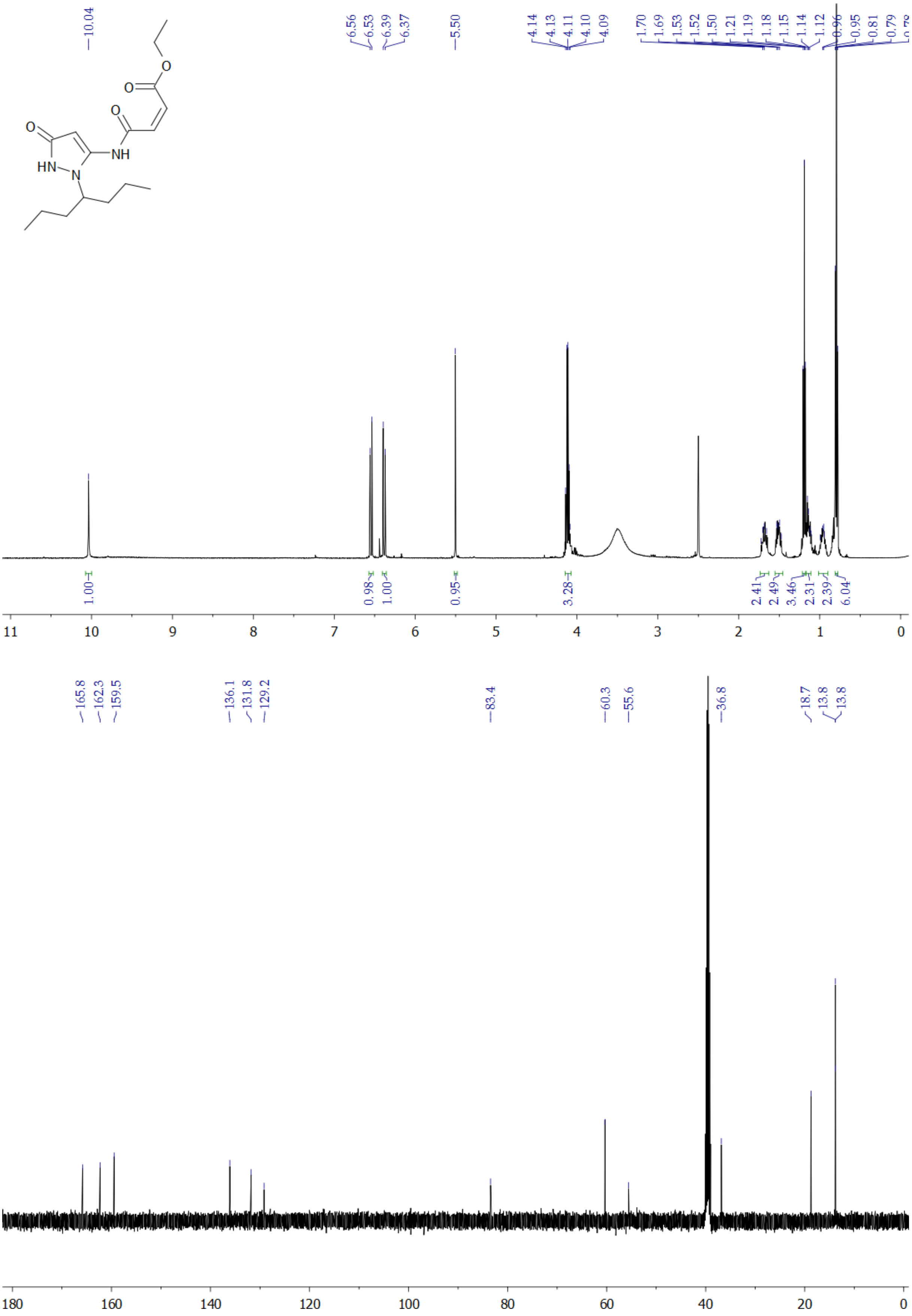

Supplementary Figure 23: ^{1}H- and ^{13}C-NMR spectrum of **17**

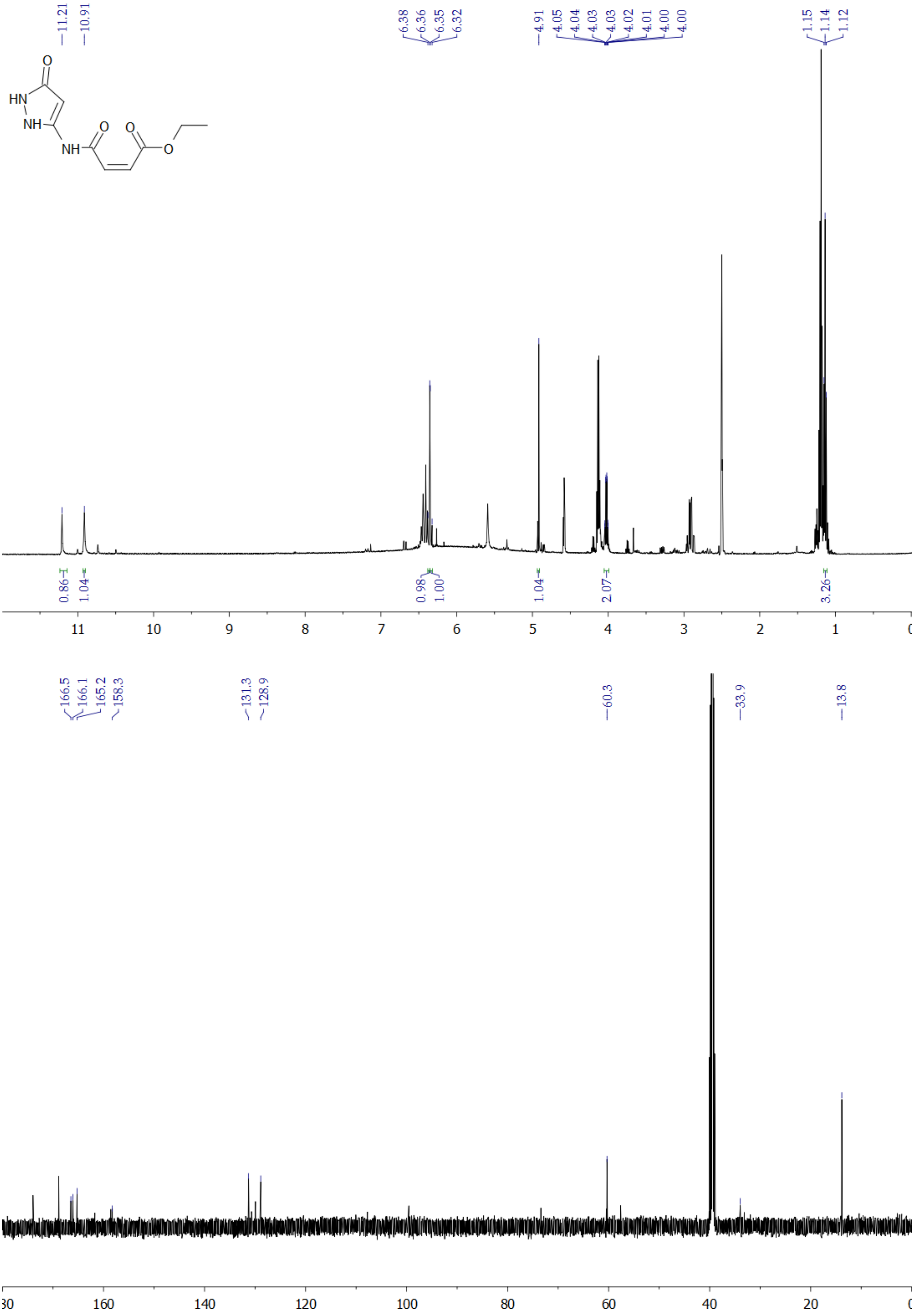

Supplementary Figure 24: ^{1}H- and ^{13}C-NMR spectrum of **18**

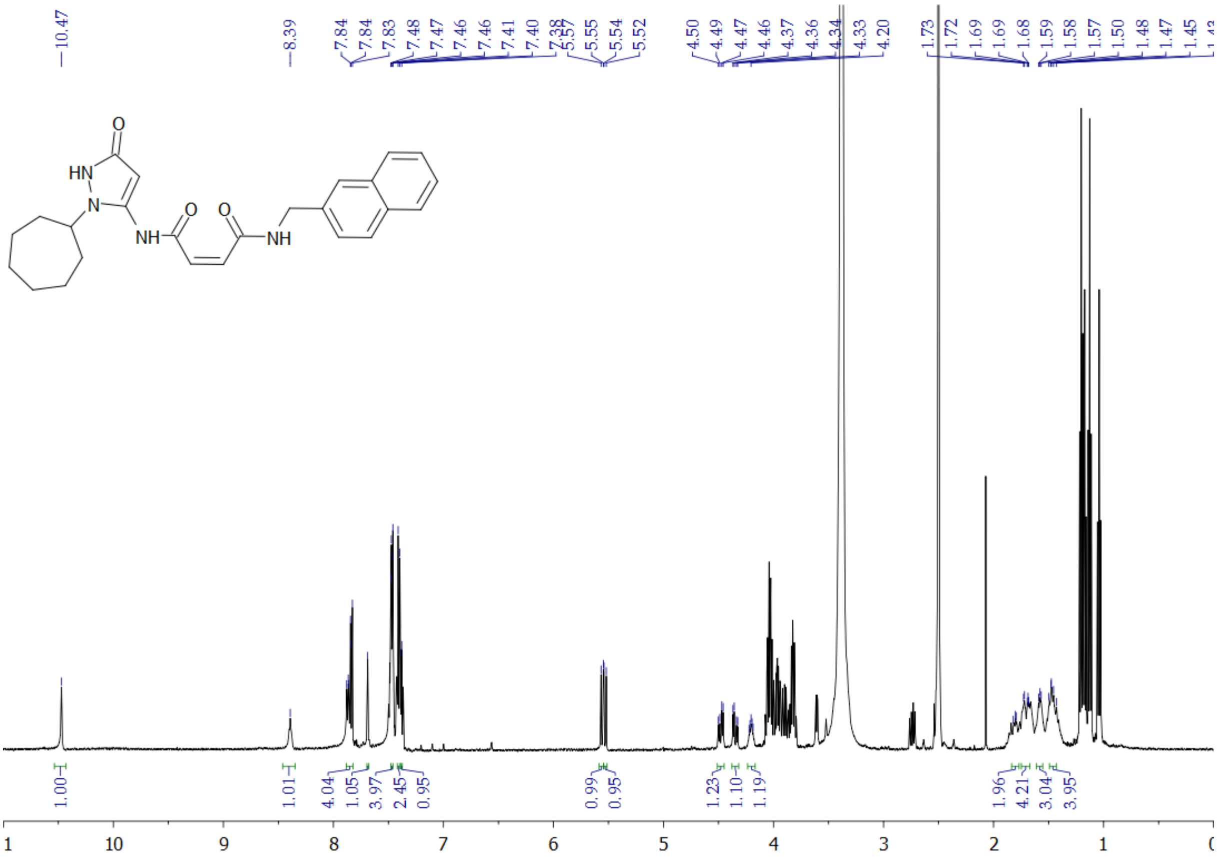

Supplementary Figure 25: ^{1}H-NMR spectrum of **19**

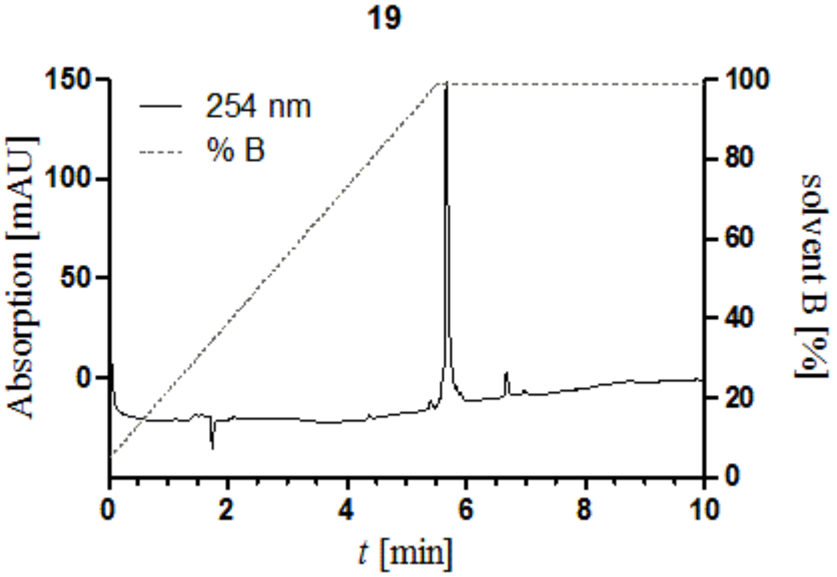

Supplementary Figure 26: RP-HPLC chromatogram of **19**

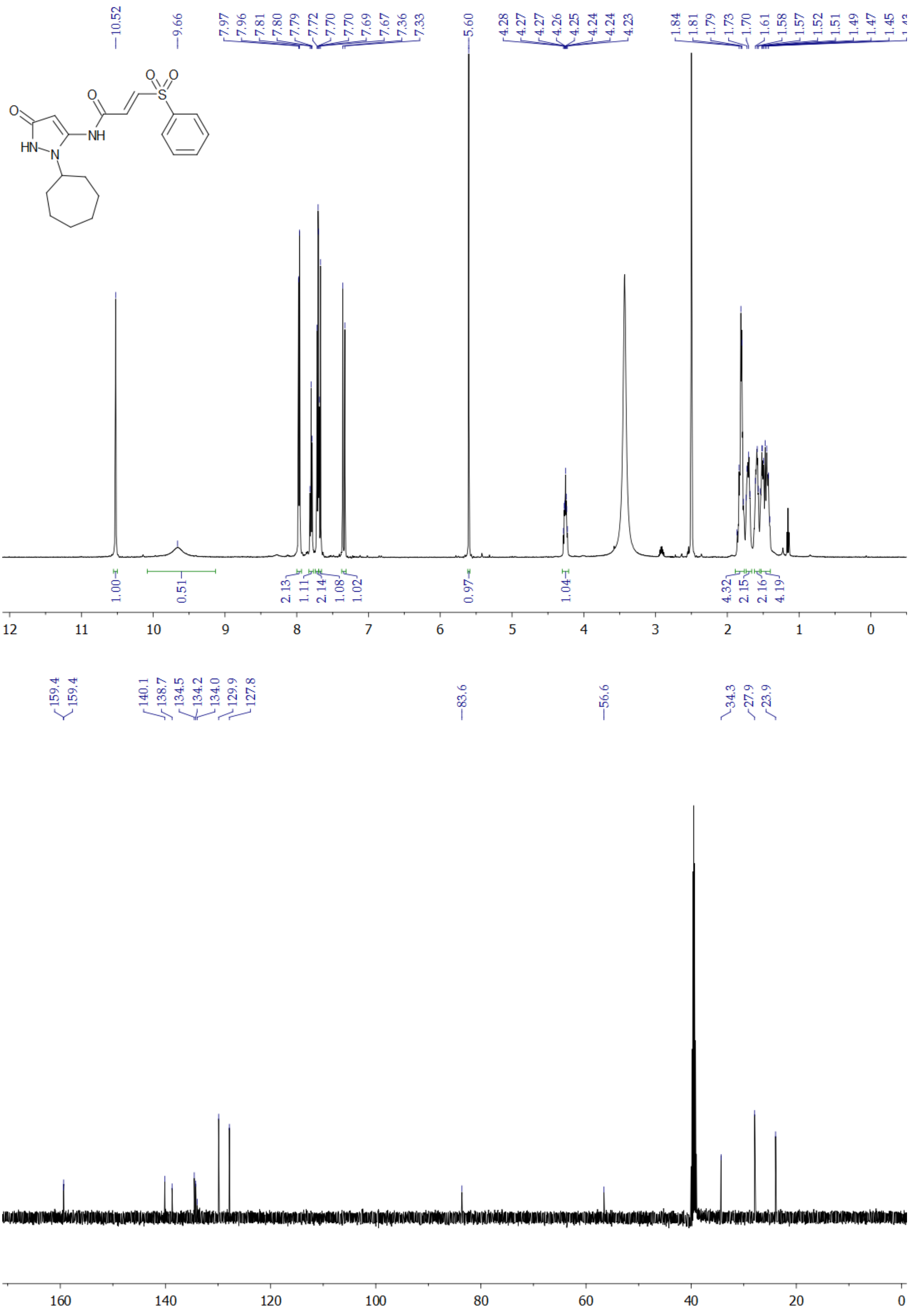

Supplementary Figure 27: ^{1}H- and ^{13}C-NMR spectrum of **20**

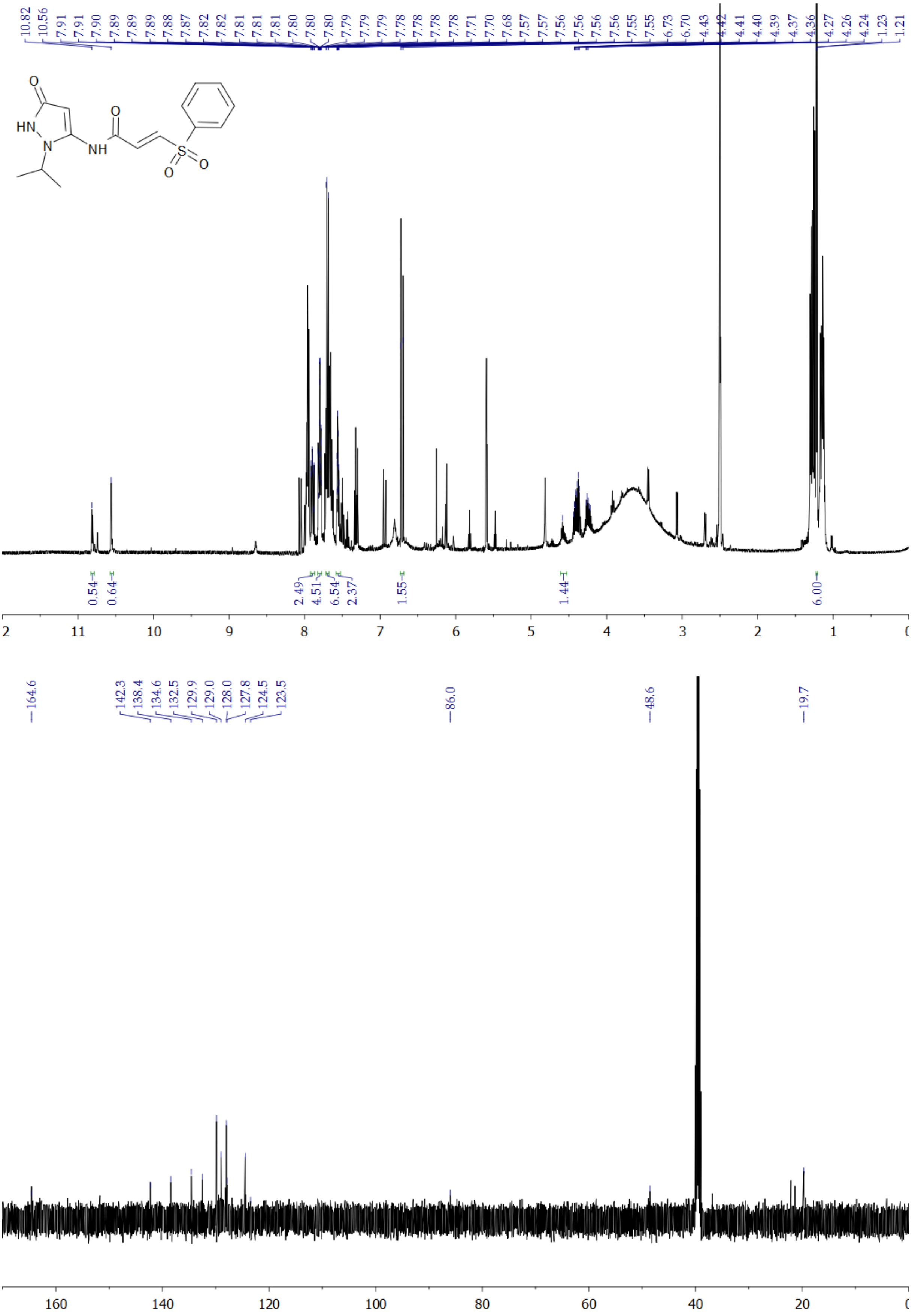

Supplementary Figure 28: ^{1}H- and ^{13}C-NMR spectrum of **21**

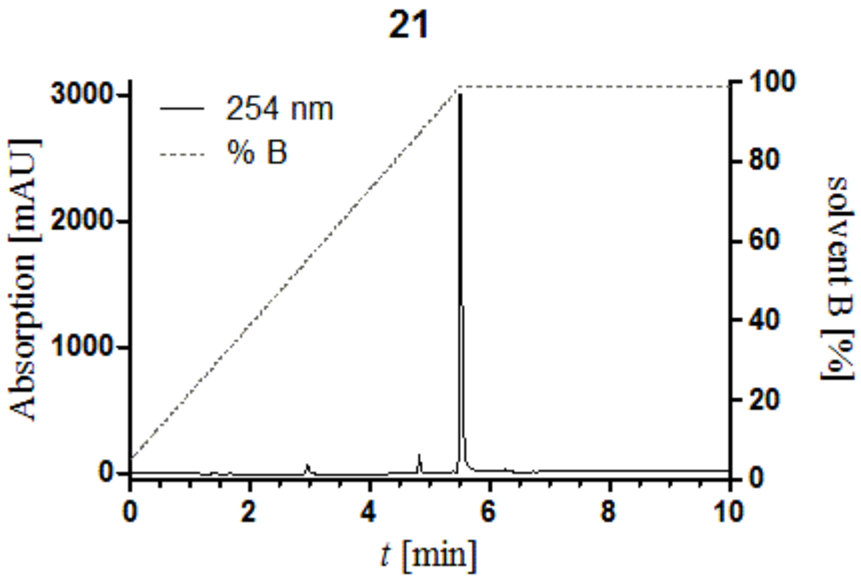

Supplementary Figure 29: RP-HPLC chromatogram of **21**

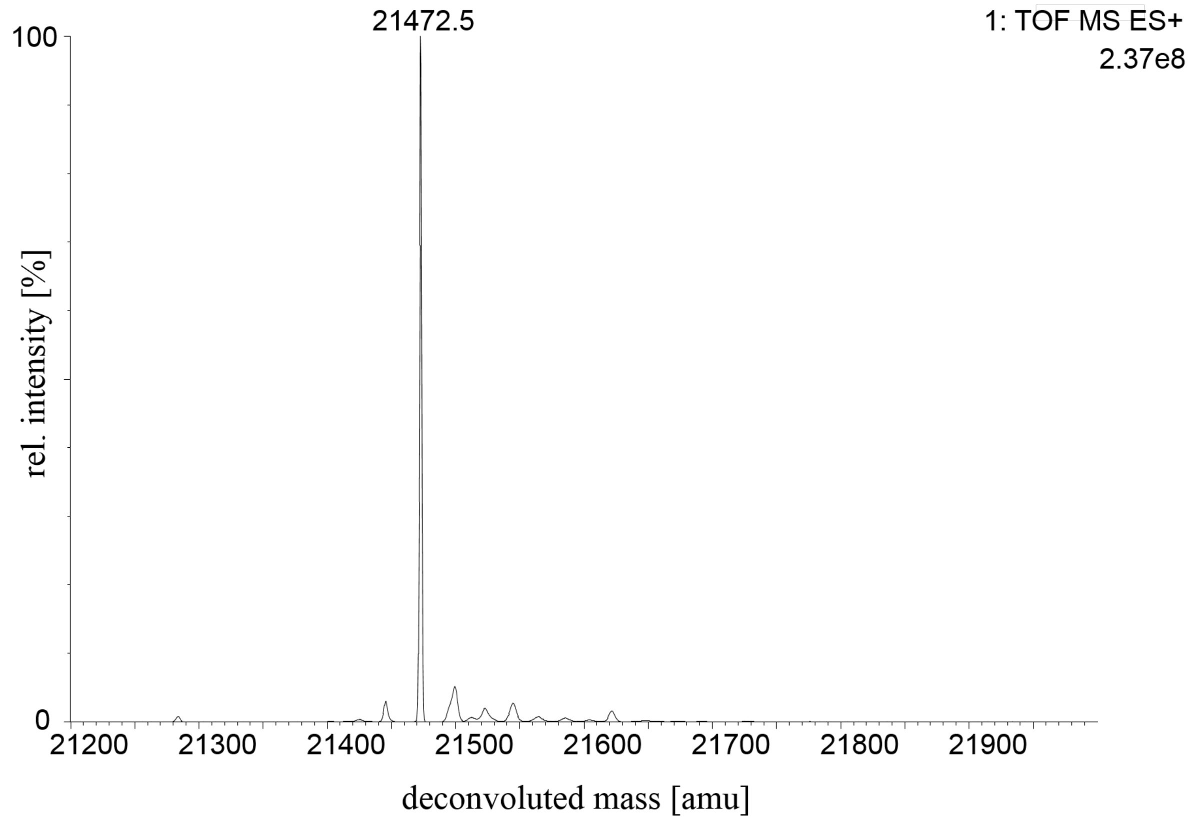

Supplementary Figure 30: Deconvoluted ESI-MS spectrum of CVB3 $3C^{Pro}$

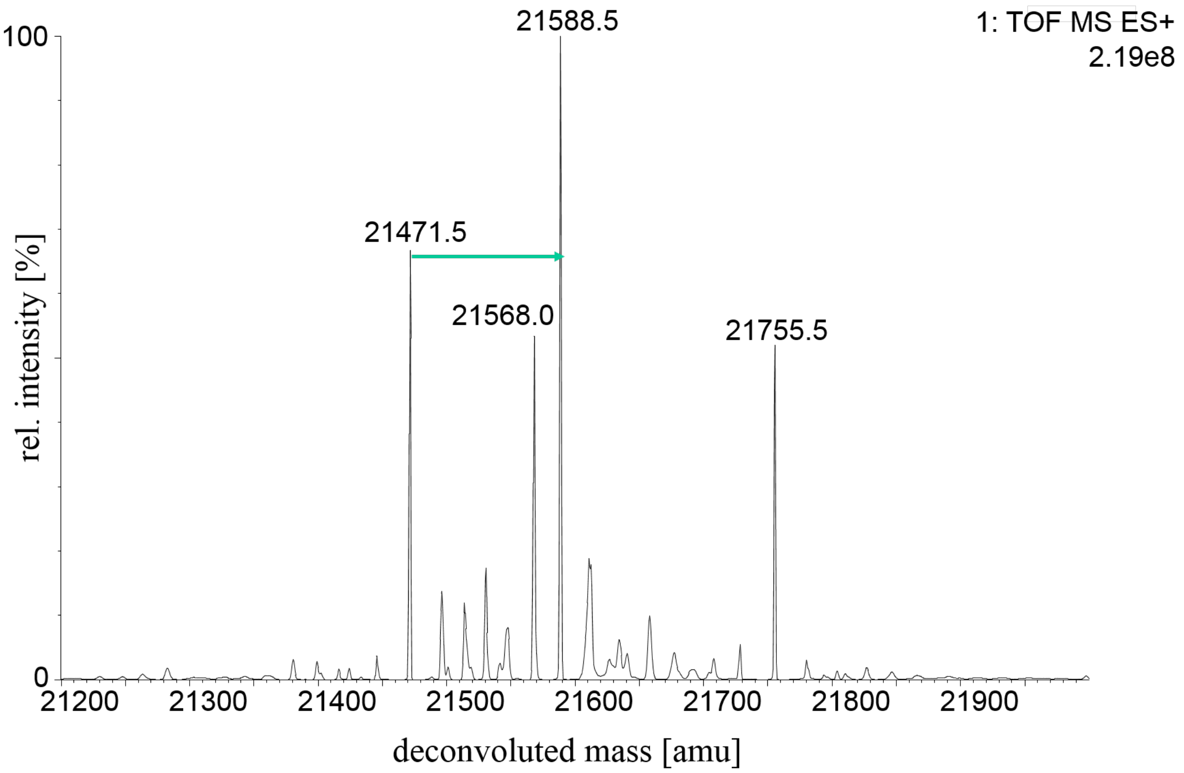

Supplementary Figure 31: Deconvoluted ESI-MS spectrum of CVB3 $3C^{Pro}$ with **1**

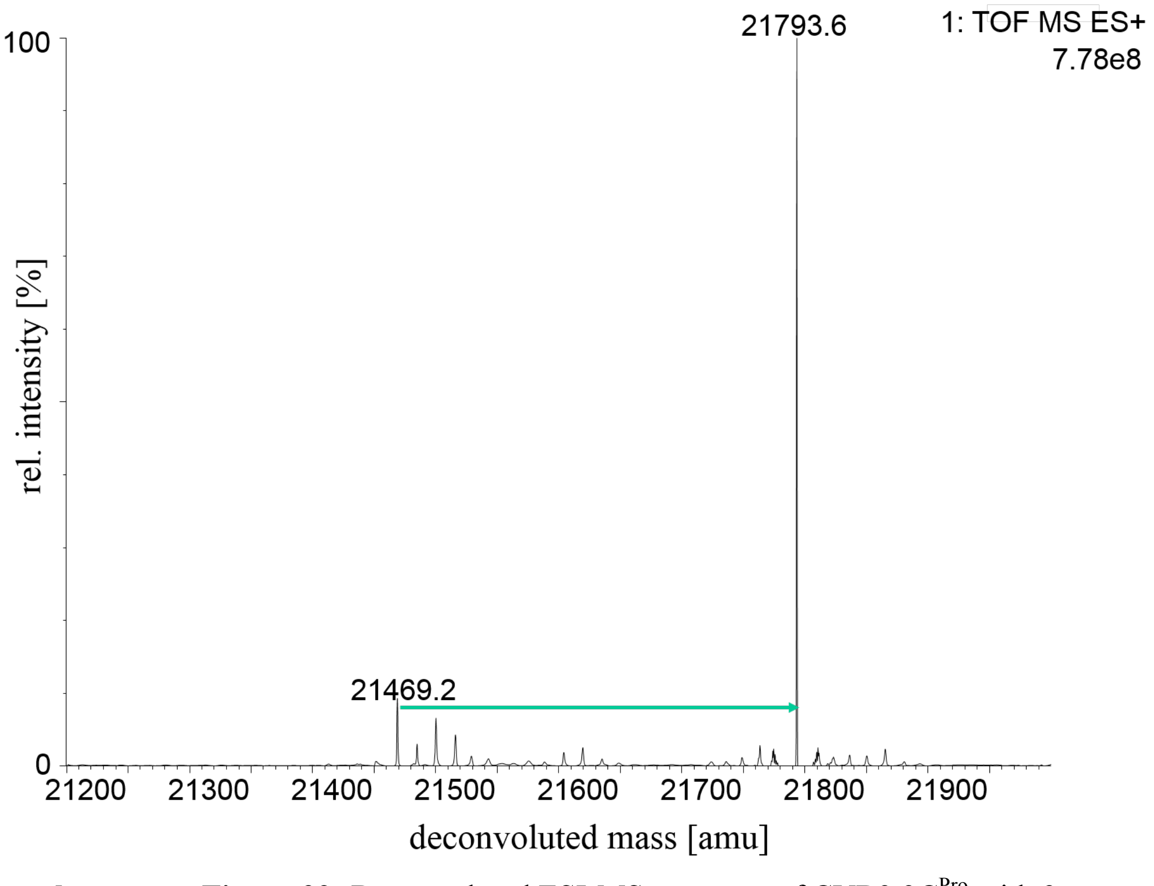

Supplementary Figure 32: Deconvoluted ESI-MS spectrum of CVB3 $3C^{Pro}$ with **3**

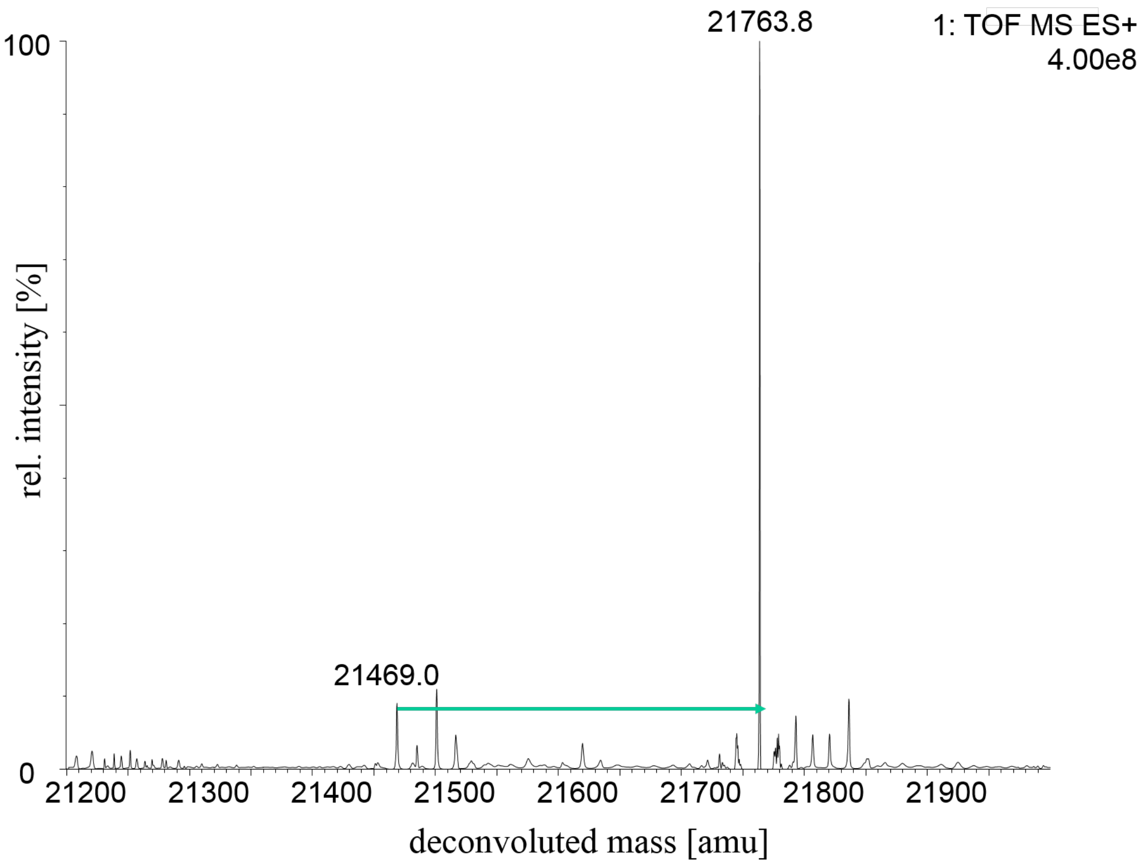

Supplementary Figure 33: Deconvoluted ESI-MS spectrum of CVB3 $3C^{Pro}$ with **10**

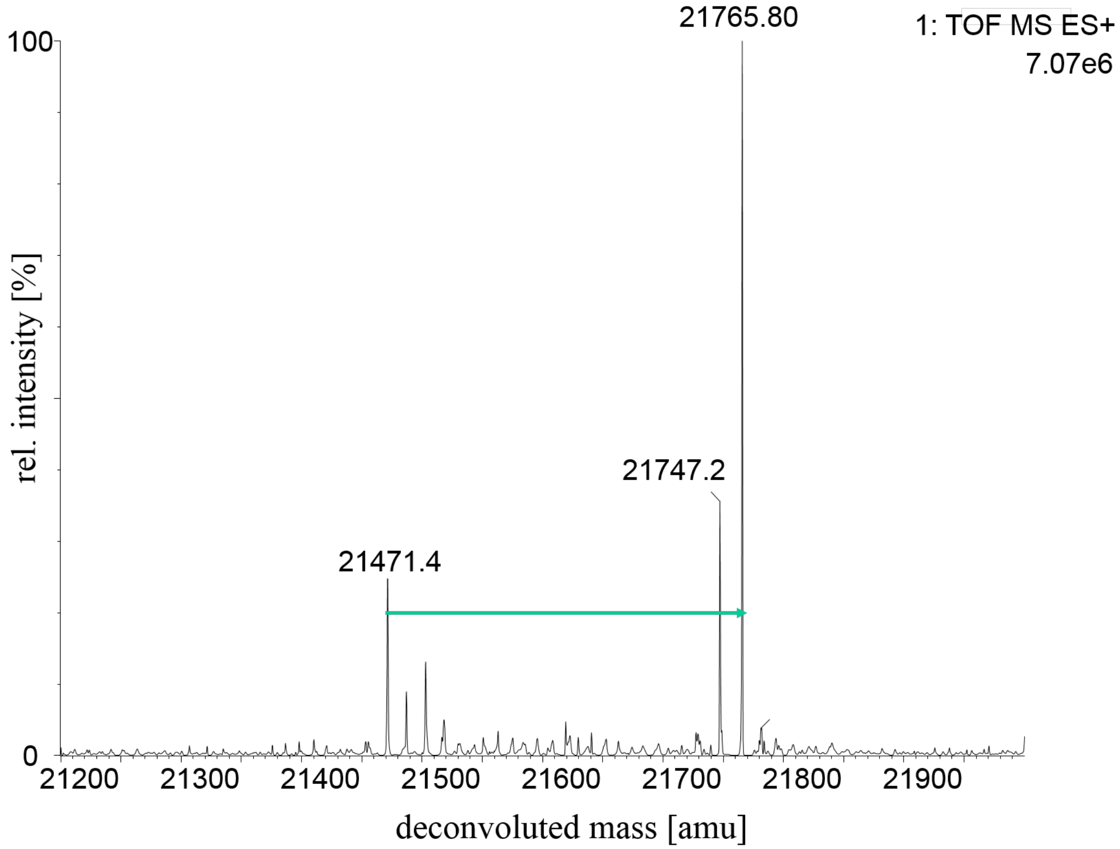

Supplementary Figure 34: Deconvoluted ESI-MS spectrum of CVB3 $3C^{Pro}$ with **12**

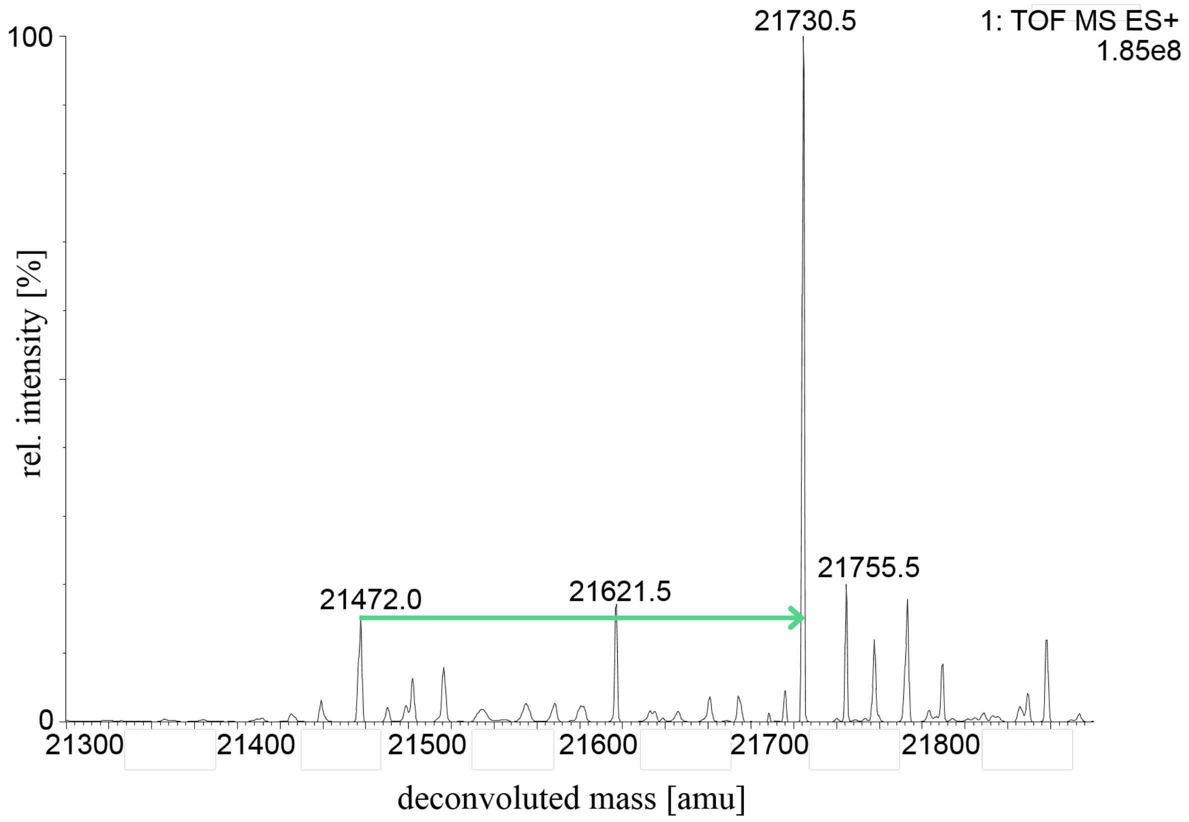

Supplementary Figure 35: Deconvoluted ESI-MS spectrum of CVB3 $3C^{Pro}$ with **20**

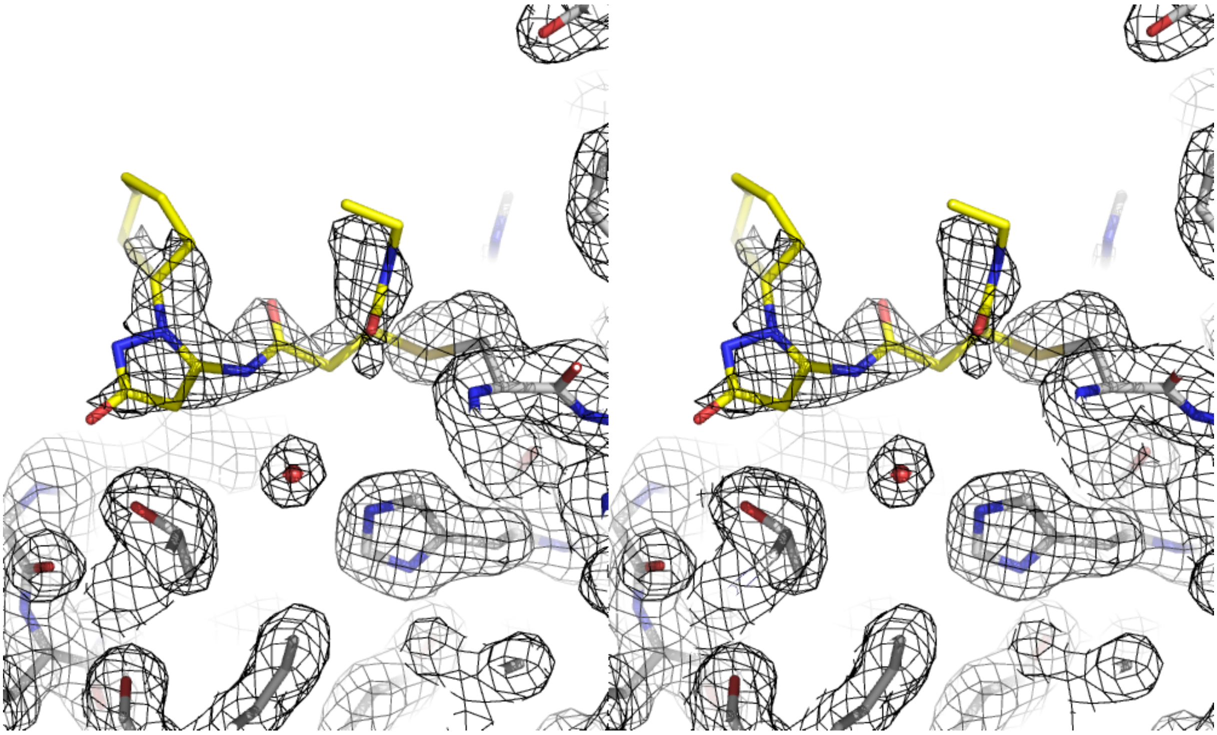

Supplementary Figure 36: Stereo image of the electron density map of the crystal structure of inhibitor **14** bound to the active site of EV-B93 3C protease (PDB: 5IYT): σÅ-weighted 2Fo-Fc electron density map, contoured at the 0.9σ level, of the EV-B93 3C protease active site showing the final refined model in sticks (protein: carbon atoms shown in grey; inhibitor **14**: carbon atoms shown in yellow).

Supplementary Tables

Supplementary Table 1.

**Compound number**	**Structure**	**k_{inact}/K_I or IC_{50} [$M^{-1}s^{-1}$] or [µM]**
Electrophilic probes		
**1**	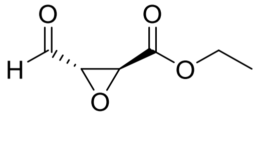	2.4 ± 0.1
Nucleophilic fragment		
**2**	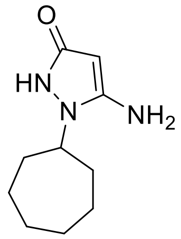	0
Epoxides		
**3**	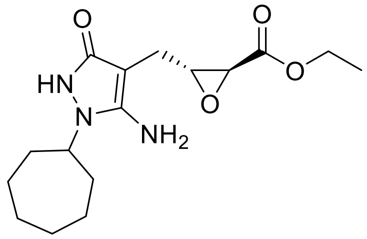	3.7 ± 0.2
**4**	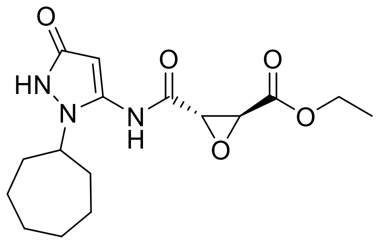	IC_{50} = 142.2 ± 5.6 µM
**5**	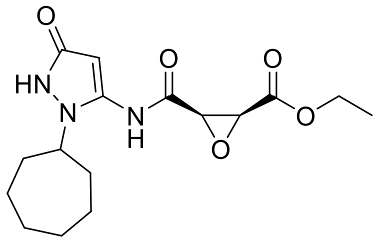	0

**6**	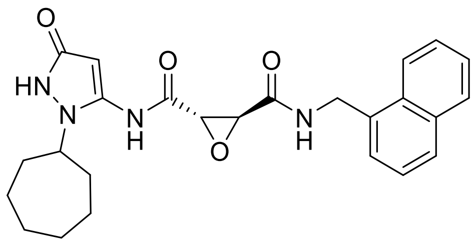	≈ 0
(E)-olefines		
**7**	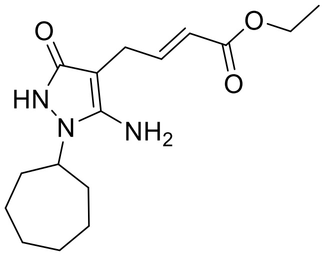	0.42 ± 0.01
**8**	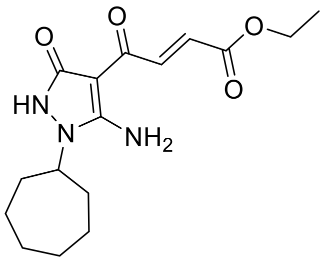	131.7 ± 4.2
**9**	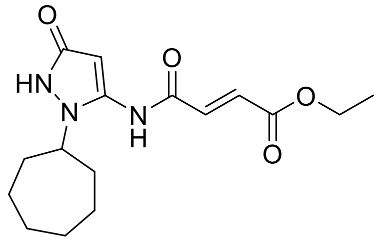	≈ 0
**10**	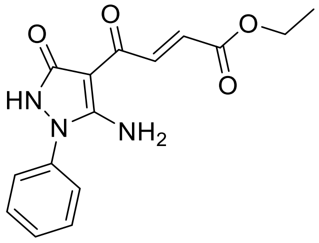	14.7 ± 0.9
**11**	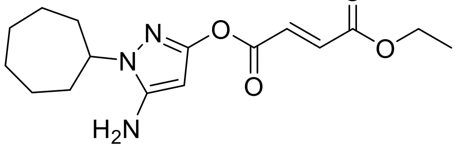	IC_{50} = 20.2 ± 1.6 µM
(Z)-olefines		

**12**	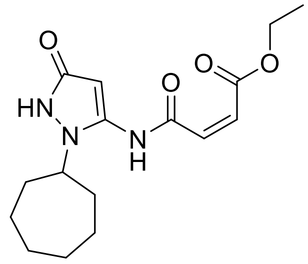	606.4 ± 24.9
**13**	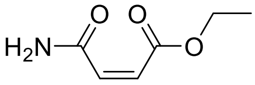	34.7 ± 1.5
**14**	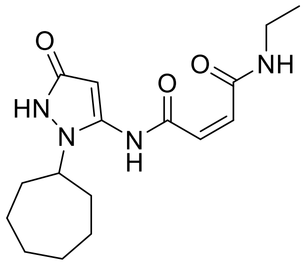	63.1 ± 3.1
**15**	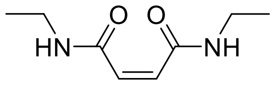	≈ 0
**16**	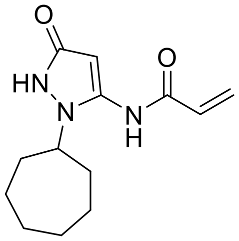	≈ 0
**17**	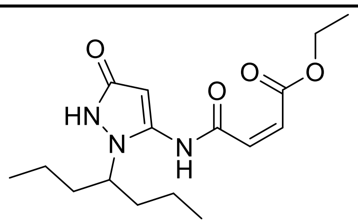	216.7 ± 30.7
**18**	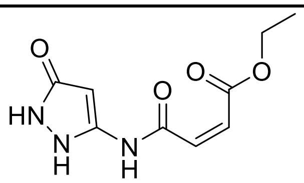	11.7 ± 0.5

**19**	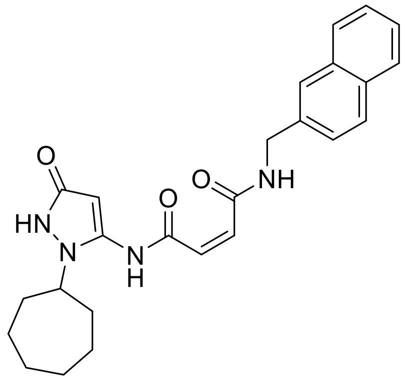	≈ 0
Vinyl sulfones		
**20**	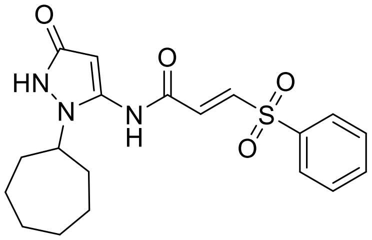	1007.7 ± 102.2
**21**	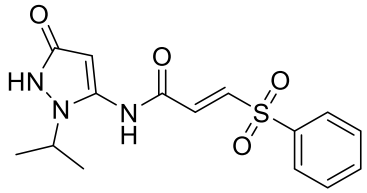	541.5 ± 6.3

Supplementary Table 2. Structures and results of docking experiments of possible ligations products of warhead **1** and fragment **2** with ID 1-7 in the active site of the CV3B 3C protease before and after covalent reaction with Cys147.

ID	Structure of putative ligationproduct	Distances [Å] between sulfur atom of Cys147 and clostest carbon atom of electrophile	Hydrogenbond count with Thr142 and His161 before reaction	Hydrogenbond count with Thr142 and His161 after reaction
1	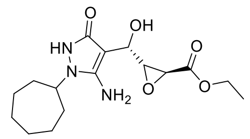	3.4	3	3
2	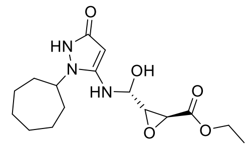	3.6	3	2
3	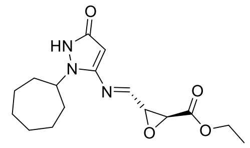	3.8	3	-
4	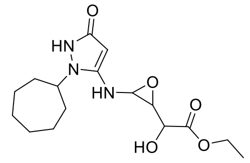	4.6	3	3
5	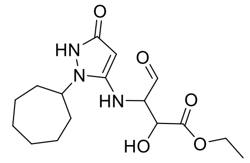	4.2	3	-
6	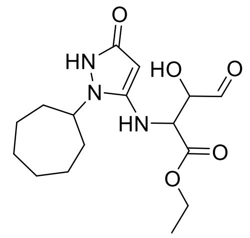	3.6	3	3
7	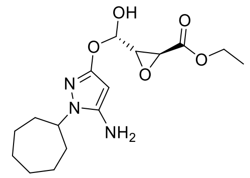	-	-	-

Supplementary Table 3. Side reactivity testing of selected compounds against different proteases (IC50 values)

Cpd#	Structure	Trypsin[1)]	Chymotrypsin[2)]	Human factor Xa[3)]	Human Caspase-3[4)]
**14**	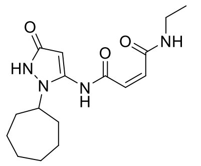	> 500 µM	> 500 µM	> 500 µM	> 500 µM
**20**	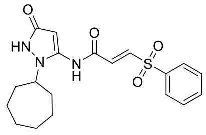	> 500 µM	> 500 µM	> 500 µM	> 300 µM

1) in 50 mM MOPS-NaOH pH 8.0 / 154 mM NaCl with 57 µM tert. butyloxycarbonyl (Boc)-LGR-7-amino-4-methylcoumarin (AMC) as substrate;
2) in 50 mM MOPS-NaOH pH 8.0 / 154 mM NaCl with 1.6 mM AF-AMC as substrate.
3) in 50 mM MOPS-NaOH pH 8.0 / 154 mM NaCl with 114 µM Boc-LGR-AMC as substrate;
4) in 50 mM HEPES-NaOH pH 7.5 / 100 mM NaCl / 1 mM EDTA / 0.1% (w/v) CHAPS / 10% (v/v) glycerol /2 mM DTT with 10 µM acetyl-DEVD-AMC as substrate;

Supplementary Methods

General synthetic information

Identity and purity (>95%) of all compounds were determined by chromatography (silica or RP-18 HPLC), by fully assigned ^{1}H- and ^{13}C NMR spectra (see supplementary NMR spectra) and by high-resolution mass spectra. Solvents were purchased in *p.a.* quality by VWR International GmbH (Darmstadt, Deutschland), Carl Roth GmbH & Co. KG (Karlsruhe, Deutschland) and Sigma Aldrich (Steinheim, Deutschland). For HPLC and MS analytics solvents with corresponding quality were used. Water was used from a Milli Q-station from Millipore (Eschborn, Germany). Only deuterated solvents from Euriso-Top GmbH (Saarbrücken, GmbH) were used for NMR spectroscopy. Chemicals were purchased from Sigma Aldrich, Carl Roth GmbH & Co. KG, Merck KGaA (Darmstadt, Germany), Alfa Aesar (Karlsruhe, Germany), ABCR GmbH & Co. KG (Karlsruhe, Germnay), and Fluka Chemie GmbH (Buchs, Switzerland) with the highest purity and used without further purification. HPLC was performed with an Agilent 1260 Infinity Binary LC System equipped with a Nucleodur C18 HTec, 5 µm by Macherey Nagel (Düren, Germany). NMR was performed with a Jeol ECP500 or a Bruker AvanceIII 700 spectrometre with deuterated solvents purchased from Eurisotop (Saint-Aubin Cedex, France).

Chromatographic methods

Purification with high performance liquid chromatography was performed with an Agilent 1260 Infinity Binary LC System equipped with a Nucleodur C18 HTec, 5 µm by Macherey Nagel (Düren, Germany). A gradient from water and acetonitrile (with 0.1% trifluoroacetic acid respectively) was utilized to separate the compounds.

Flash chromatography was performed with an Isolera One System from Biotage (Upsala, Sweden) using pre-packed KP-Sil SNAP-cartridges.

HPLC-ESI-MS spectra were recorded with an Agilent 1100 LC/MS system equipped with a Luna-C18, 3 µm by Phenomenex. A gradient of water (solvent A) and acetonitrile (solvent B) was used and each solvent contained 0.1% (vol/vol) formic acid. During the analysis the the solvent ratio (A:B) was changed from 95:5 to 1:99, while maintaining a flowrate of 1 ml/min.

Mass spectrometry with $3C^{Pro}$-C147A mutant

20 µL of a 0.425 µg/ml enzyme solution (containing also 100 mM Tris*HCl, 200 mM NaCl, 4 mM EDTA, 20% glycerine) were incubated with 250 µM inhibitor for 60 minutes. Afterwards 10 µL of this solution were handled as stated in the *mass spectrometry of protein-inhibitor samples* part.

Chemical synthesis and analysis

Ethyl (2*S*,3*S*)-epoxy-4-oxo-butanoate 1

The electrophilic probe **1** has been synthesized according to the procedure of Marigo *et al.*[1]

5-Amino-1-cycloheptyl-1,2-dihydro-pyrazol-3-one 2[2]

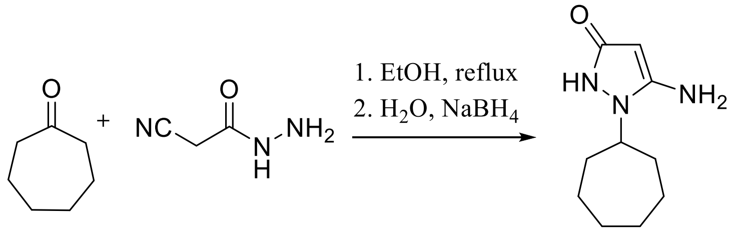

2

A mixture of 2-cyanoacetyl hydrazide (1.25 g, 12.7 mmol) and cycloheptanone (1.42 g, 12.7 mmol) was heated in ethanol (12 ml) under reflux for 1 h. After cooling, the precipitate was filtered off, washed with cold ethanol, recrystallized from ethanol and dried under vacuum. The solid intermediate, 2-cyano-*N*-cycloheptylidene acetylhydrazide, was placed in a big flask and suspended with water (120 ml). With vigorous stirring sodium borohydride (265 mg, 7 mmol, 0.55 eq) was added and the suspension was stirred for 16 h. The solids were filtered off, washed thoroughly with water and lyophilized from dioxane/water yielding the pyrazolone **2** as a white solid.

Yield: 1.91 g (77%); ESI-HRMS (*m/z*): $[M+Na]^+$ calc. for $C_{10}H_{17}N_3NaO^+$, 218.1264 Da; found, 218.1258 Da; ^{1}H-NMR (300 MHz, DMSO-d_6): δ [ppm] = 8.79 (bs, -N*H*, 1H), 5.61 (s, -NH_2, 2H), 4.31 (s, COC*H*, 1H), 3.86 (m, NC*H*(CH_2)$_2$, 1H), 1.69-1.38 (m, CH_2, 12H); ^{13}C-NMR (75 MHz, pyridine-d_5): d [ppm] = 166.0, 152.5, 77.0, 58.5, 34.1 (2), 28.7 (2), 25.4 (2); IR (ATR): $\bar{\nu}$ = 3112 (w, br, *N*-H), 2927 (w, =C-H), 2857 (w, -C-H), 1594 (s, C=C-**C=O**), 1558 (s, **C=C**-C=O), 1501, 1449, 1004 (m, =C-H) cm^{-1}; mp: > 223°C (decomp.)

O-Ethyl 4-[(5-amino-1-cycloheptyl-1,2-dihydropyrazol-3-one-4-yl]-(2*S*,3*R*)-epoxy-butanoate 3

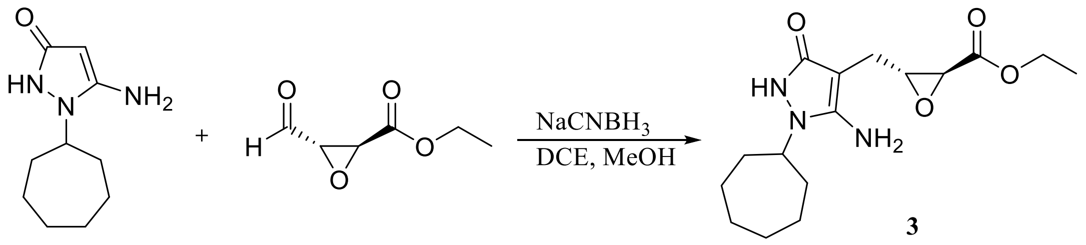

3

Ethyl (2*S*,3*S*)-epoxy-4-oxo-butanoate (50 mg, 0.34 mmol, 1 eq) was dissolved in 1,2-dichloroethane (5 ml). After addition of **2** (50 mg, 0.25 mmol, 0.75 eq) in methanol (1 ml), sodium cyanoborohydride (62 mg, 1 mmol, 3 eq) was added and the solution was stirred for three hours. The solvent was removed in vacuo and the residue was purified with reversed phase high performance liquid chromatography, yielding **3** as a colorless solid.

Yield: 48 mg (45%); ESI-HRMS (*m/z*): $[M+Na]^+$ calc. for $C_{16}H_{25}N_3NaO_4^+$, 324.1918 Da; found, 324.1927 Da; ^{1}H-NMR (500 MHz, CD_3CN): δ [ppm] = 4.16 (qd, $^3J_{H\text{-}H}$ = 7.1 Hz, 3.7 Hz, COOC*H*$_2$CH$_3$, 2H), 4.10 (m, NC*H*(CH$_2$)$_2$, 1H), 3.23 (m, C*H*OC*H*COOEt, 2H), 2.70 (dd, $^3J_{H\text{-}H}$ = 16.0 Hz, 3.6 Hz, NHCOCC*H*$_2$, 1H), 2.55 (dd, $^3J_{H\text{-}H}$ = 16.0 Hz, 3.6 Hz, NHCOCC*H*$_2$, 1H), 1.94 – 1.89 (m, cycloheptyl-C*H*$_2$, 2H), 1.79 – 1.72 (m, cycloheptyl-C*H*$_2$, 4H), 1.65 – 1.63 (m, cycloheptyl-C*H*$_2$, 2H), 1.60 – 1.51 (m, cycloheptyl-C*H*$_2$, 4H), 1.23 (t, $^3J_{H\text{-}H}$ = 7.1 Hz, COOCH$_2$C*H*$_3$, 3H); ^{13}C-NMR (125 MHz, CD_3CN): δ [ppm] = 169.6, 163.6, 160.7, 152.5, 829, 62.3, 59.9, 57.7, 53.2, 33.2 (2), 28.1 (2), 25.0 (2), 22.8, 14.4; mp: 90-91°C.

O-Ethyl 4-(1-cycloheptyl-1,2-dihydropyrazol-3-one-5-yl)-amino-4-oxo-(2*R*,3*R*)/(2*S*,3*S*)-epoxy-butanoate 4

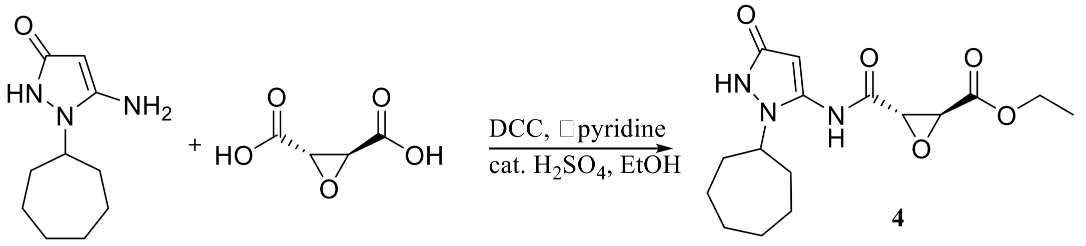

2 (1 eq) was dissolved in pyridine (10 ml per 0.5 mg **2**) and stirred vigorously. Trans-2,3-Epoxysuccinic acid (2.95 eq) and *N,N'*-dicyclohexylcarbodiimide (2.95 eq) was added and the suspension stirred overnight. The solvent was removed in vacuo, the residue was taken up in ethanol and aqueous sodium hydroxide solution (2 mol/l, 2 eq) was added dropwise. After 30 minutes of stirring, the solvent was removed in vacuo, after which the residue was taken up in aqueous acetonitrile, filtered and purified with reversed phase liquid chromatography, which yielded 4-(1-cylcoheptyl-1,2-dihydropyrazol-3-one-5-yl)-amino-4-oxo-epoxybutanoic acid as a colorless intermediate. This was dissolved in ethanol, mixed with two drops of concentrated sulfuric acid and stirred at 90°C for 60 minutes. After cooling, the solvent was removed in vacuo and the residue purified with reversed phase liquid chromatography. Depending on the configuration of the employed epoxysuccinic acid, an enantiomeric mixture of (2R,3R)/(2S,3S) **4** from *trans*-epoxysuccinic acid and (2R,3S)/(2S,3R) **5** from *cis*-epoxysuccinic acid was obtained.

Yield: 48.8 mg (62%); ESI-MS (*m/z*): $[M+H]^+$ calc. for $C_{16}H_{24}N_3O_5^+$, 338.2 Da; found, 338.2 Da; ^{1}H-NMR (300 MHz, DMSO-d_6): δ [ppm] = 10.34 (s, NN*H*CO, 1H), 10.02 (s, CN*H*CO, 1H), 5.48 (s, NHCOC*H*, 1H), 4.21 (q, $^3J_{H\text{-}H}$ = 7.2 Hz, CH_3CH_2, 2H), 4.14 (m, C*H*$(CH_2)_2$, 1H), 3.92 (d, $^3J_{H\text{-}H}$ = 1.8 Hz, NHCOC*H*OCHCOOEt, 1H), 3.73 (d, $^3J_{H\text{-}H}$ = 1.8 Hz, NHCOCHOC*H*COOEt, 1H), 1.86-1,40 (m, cycloheptyl-CH_2, 12H), 1.25 (t, $^3J_{H\text{-}H}$ = 7.1 Hz, CH_3CH$_2$, 3H); ^{13}C-NMR (75 MHz, DMSO-d_6): δ [ppm] = 167.0, 163.7, 159.2, 133.4, 84.2, 61.6, 56.5, 53.0, 51.5, 34.2 (2), 27.9 (2), 23,9 (2), 13.9; mp.: > 250°C (decomp.).

O-Ethyl N-(1-cycloheptyl-1,2-dihydropyrazol-3-one-5-yl)-3-carbamoyl-(2*R*,3*S*)/(2*S*,3*R*)-epoxy-butanoate 5

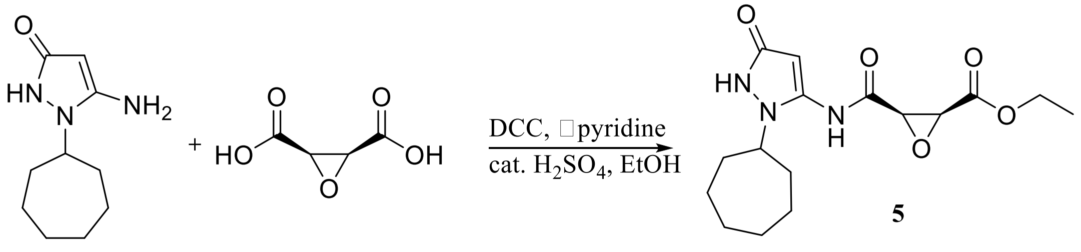

Yield: 43.8 mg (25%); ESI-HRMS (*m/z*): $[M+H]^+$ calc. for $C_{16}H_{24}N_3O_5^+$, 338.1710 Da; found, 338.1734 Da; ^{1}H-NMR (500 MHz, DMSO-d_6): δ [ppm]= 10.13 (s, NN*H*CO, 1H), 9.58 (s, CN*H*CO, 1H), 5.30 (s, NNHCOC*H*, 1H), 4.13 (q, $^3J_{H\text{-}H}$ = 7.1 Hz, CH_3CH_2, 2H), 4.11 (m, C*H*(CH_2), 1H), 3.99 (s, NHCOC*H*OC*H*COOEt, 2H), 1.81-1.42 (m, cycloheptyl-CH_2, 12H), 1.18 (t, $^3J_{H\text{-}H}$ = 7.1 Hz, CH_3CH$_2$, 3H); ^{13}C-NMR (125 MHz, DMSO-d_6): δ [ppm]= 166.1, 163.8, 160.5, 134,8, 84.8, 61.1, 56.4, 53.7, 52.4, 34.3, 34.2, 27.8 (2), 23.9 (2), 13.9; mp.: 70-71°C.

N-(1-Cycloheptyl-1,2,5-dihydro-1*H*-pyrazol-3-one-5-yl)-*N′*-(naphth-1-yl)- (2*R*,3*R*)/(2*S*,3*S*)-epoxy-butanediamide 6

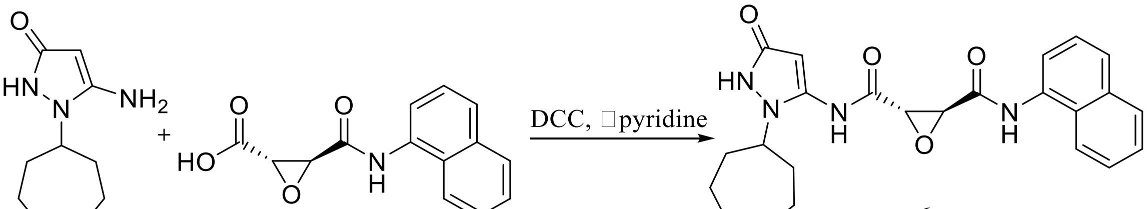

6

Trans-epoxysuccinic acid (=2,3-epoxybutanediacid, 200 mg, 1.5 mmol, 1.25 eq) and *N*,*N*'-dicyclohexylcarbodiimide (258 mg, 1.25 mmol, 1 eq) were dissolved in dry *N*,*N*-dimethylformamide (15 ml). 1-Naphthylmethylamine (183 µl, 1.25 mmol, 1 eq) was added dropwise and the solution stirred until a white precipitate was formed. The suspension was filtered and the filtrate dried in vacuo. The residue was passed through a short pad of silica using an eluent consisting of methylene chloride and methanol (DCM:MeOH, 9:1 v/v). This furnished *N*-naphthylmethyl-(2R,3R)/(2S,3S)-epoxysuccinamic acid (240 mg, 0.88 mmol, 70%) as a white solid.

This intermediate was dissolved in pyridine (10 ml) and **2** (86 mg, 0.44 mmol, 0.5 eq) and *N*,*N*'-dicyclohexylcarbodiimide (181 mg, 0.88 mmol, 1eq) was added. The resulting suspension was stirred for 16 hours after which it was filtered and dried in vacuo. The residue was washed with toluene and remaining solvents were coevaporated with toluene. The dried residue was dissolved in ethanol and sodium hydroxide (17.5 mg, 0.44 mmol, 1 eq) was added. After stirring for one hour the suspension was filtered, dried in vacuo and the residue subjected to normal phase flash chromatography using an hexane-ethylacetat gradient (1:0 → 0:1) as eluent, furnishing **6** as a white solid.

Yield: 18 mg (10%); ESI-MS (*m/z*): $[M+H]^+$ calc. for $C_{22}H_{29}N_4O_4^+$: 449.2 Da, found: 449.3 Da; ^{1}H-NMR (500 MHz, DMSO-d_6): δ [ppm]= 10.33 (s, ArN*H*COCH, 1H), 9.62 (s, NN*H*COCHC, 1H), 9.09 (t, $^3J_{H-H}$ = 5.8 Hz, CON*H*CH_2, 1H), 8.08 (dd, $^3J_{H-H}$ = 8.3, 1.3 Hz,

Ar*H*, 1H), 7.97 (dd, $^3J_{H\text{-}H}$ = 7.8, 1.9 Hz, Ar*H*, 1H), 7.88 (dd, $^3J_{H\text{-}H}$ = 6.5, 3.1 Hz, Ar*H*, 1H), 7.57 (m, Ar*H*, 2H), 7.49 (m, Ar*H*, 2H), 5.47 (s, NNHCOC*H*C, 1H), 4.81 (d, $^3J_{H\text{-}H}$ = 5.7 Hz, NHCH_2Naphthylmethyl, 2H), 4.16 (tt, $^3J_{H\text{-}H}$ = 9.0, 5.3 Hz, NC*H*(CH_2)$_2$, 1H), 3.89 (d, $^3J_{H\text{-}H}$ = 1.8 Hz, Oxirane-C*H*, 1H), 3.70 (d, $^3J_{H\text{-}H}$ = 1.8 Hz, Oxirane-C*H*, 1H), 1.88 – 1.63 (m, cycloheptyl-CH_2, 6H), 1.58 (m, cycloheptyl-CH_2, 2H), 1.55 – 1.37 (m, cycloheptyl-CH_2, 4H); ^{13}C-NMR (125 MHz, DMSO-d_6): δ [ppm]= 165.9, 164.9, 134.2, 134.0, 133.8, 131.3, 129.1, 128.4, 126.9, 126.4, 125.9, 123.9, 57.0, 53.5, 52.9, 40.9, 34.7 (2), 28.3 (2), 24.4 (2) two carbonyl atoms and C-4 could not be resolved. mp.: 141-142°C.

O-Ethyl 4-(5-Amino-1-cycloheptyl-1,2-dihydropyrazol-3-one-4-yl)-2E-butenoate 7

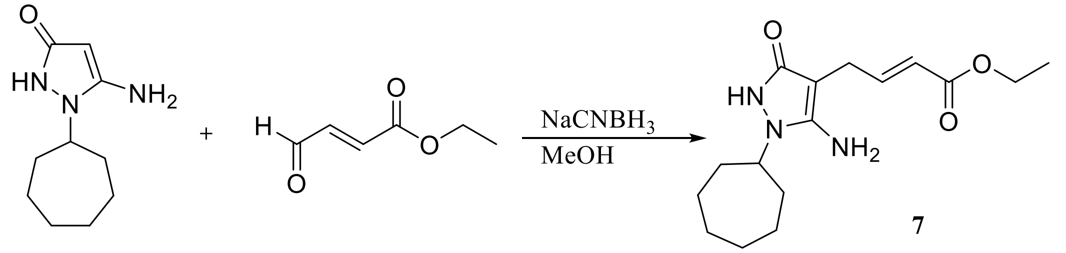

Ethyl (2E)-4-oxo-2-butenoate (62 µl, 0.5 mmol, 1 eq) was dissolved in methanol (15 ml). After addition of **2** (100 mg, 0. 5 mmol, 0.1 eq) sodium cyanoborohydride (62 mg, 1 mmol, 2 eq) was added and the solution was stirred for twelve hours. The reaction was quenched with water, the solvent removed in vacuo and the residue was purified with reversed phase high performance liquid chromatography, yielding **7** as a white solid.

Yield: 32 mg (20%); ESI-HRMS (*m/z*): $[M+H]^+$ calc. for $C_{16}H_{26}N_3O_3^+$, 308.1969 Da; found, 308.1993 Da; ^{1}H-NMR (700 MHz, CD_3CN): δ [ppm] = 6.88 (dt, $^3J_{H\text{-}H}$ = 15.6, 5.8 Hz, C*H*CHCOOEt, 1H), 5.76 (dt, $^3J_{H\text{-}H}$ = 15.6, 1.8 Hz, CHC*H*COOEt, 1H), 5.31 (s, NNHCOC*H*C, 1H), 4.13 (q, $^3J_{H\text{-}H}$ = 7.1 Hz, CH_2CH$_3$, 2H), 4.11 – 4.06 (m, NC*H*(CH_2)$_2$, 1H), 3.12 (dd, $^3J_{H\text{-}H}$ = 5.8, 1.8 Hz, CH_2CHCHCOOEt, 2H), 1.93 – 1.89 (m, cycloheptyl-CH_2, 4H), 1.80 – 1.72 (m, cycloheptyl-CH_2, 4H), 1.68 – 1.60 (m, cycloheptyl-CH_2, 2H), 1.60 – 1.47 (m, cycloheptyl-CH_2, 2H), 1.23 (t, $^3J_{H\text{-}H}$ = 7.1 Hz, COOCH$_2$CH_3, 3H); ^{13}C-NMR (175 MHz,

CD_3CN): δ [ppm] = 167.0, 161.5, 152.4, 146.1, 122.4, 84.4, 61.1, 59.9, 33.1 (2), 28.2 (2), 25.1 (2), 23.4, 14.6; mp.: 89-90°C.

O-Ethyl 4-(5-amino-1-cycloheptyl-1,2-dihydropyrazol-3-one-4-yl)-4-oxo-2E-butenoate 8

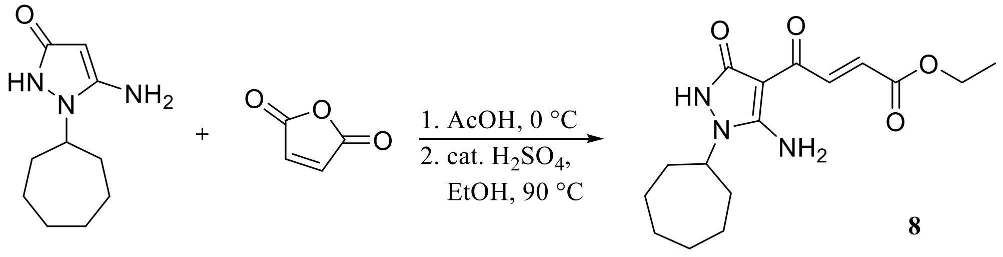

8

2 (120 mg, 0.61 mmol, 1 eq) was dissolved in ice cooled acetic acid (5 ml) maleic anhydride (120 mg, 1.22 mmol, 2 eq) was added and the solution stirred overnight. The solvent was removed in vacuo and the residue purified with flash chromatography (dichloromethane:methanol, 95:5, *v*/*v*) which yielded (E)-4-(5-amino-1-cycloheptyl-3-oxo-1,2-dihydropyrazol-4-yl)-4-oxo-2-butenoic acid as a side product. The carboxylic acid was dissolved in ethanol (5 ml) and mixed with two drops of concentrated sulfuric acid. The solution was heated to 90°C for 60 minutes. Removal of the solvent in vacuo and purification with reversed phase high performance liquid chromatography furnished **8** as a yellow solid.

Yield: 25 mg (12%); ESI-MS (*m/z*): $[M+H]^+$ calc. for $C_{16}H_{24}N_3O_4^+$, 322.1 Da; found, 322.1 Da; TLC (dichloromethane:methanol, 95:5): $R_f = 0.3$; ^{1}H-NMR (300 MHz, pyridine-d_5): δ [ppm] = 11.60 (s, NN*H*CO, 1H), 9.16 (d, $^3J_{H\text{-}H}$ = 15.6 Hz, CCOC*H*CHCOOEt, 1H), 8.36 (s, NH_2, 2H), 7.39 (d, $^3J_{H\text{-}H}$ = 15.6 Hz, CCOCHC*H*COOEt, 1H), 4.35 (tt, $^3J_{H\text{-}H}$ = 9.4 Hz, 4.9 Hz, NC*H*$(CH_2)_2$, 1H), 4.20 (q, $^3J_{H\text{-}H}$ = 7.1 Hz, COOC$H_2$$CH_3$, 2H), 2.16 – 1.89 (m, cycloheptyl-CH_2, 4H), 1.72 – 1.55 (m, cycloheptyl-CH_2, 2H), 1.47 – 1.30 (m, cycloheptyl-CH_2, 4H), 1.28 (m, cycloheptyl-CH_2, 2H), 1.14 (t, $^3J_{H\text{-}H}$ = 7.1 Hz, COOCH_2CH_3, 3H); ^{13}C-NMR (75 MHz, pyridine-d_5): δ [ppm] = 181.2, 166.6, 165.4, 161.5, 141.0, 128.3, 95.3, 60.8, 57.5, 33.2 (2), 28.2 (2), 24.7 (2), 14.2.

O-Ethyl N-(1-cycloheptyl-1,2-dihydropyrazol-3-one-5-yl)-3-carbamoyl-2E-butenoate 9

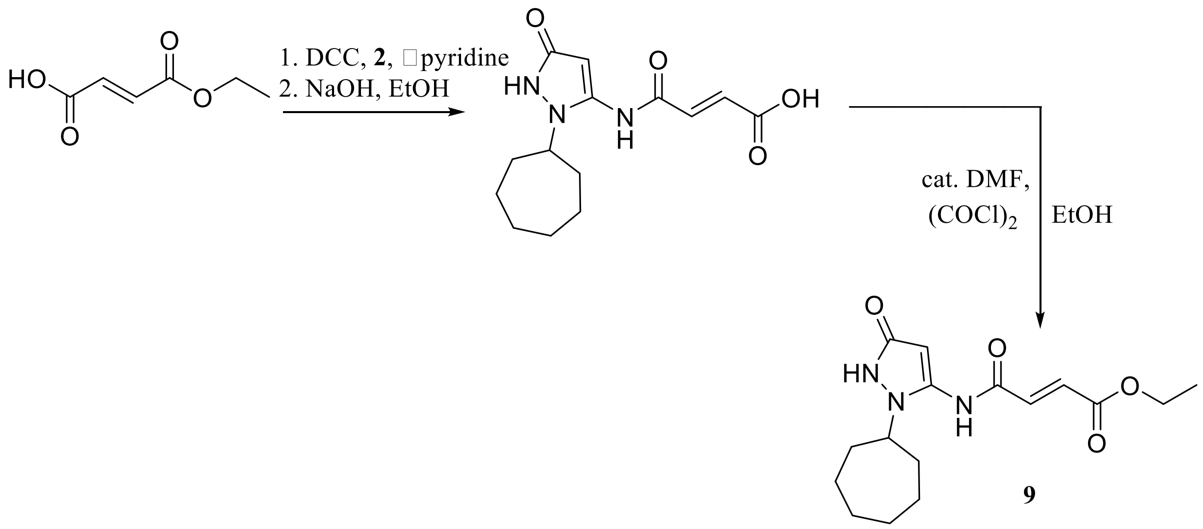

2 (66 mg, 0.34 mmol, 1 eq) was dissolved in pyridine (3 ml) and *N,N*'-dicyclohexylcarbodiimide (206 mg, 1 mmol, 3 eq) and fumaric acid monoethyl ester (143 mg, 1 mmol, 3 eq) were added. After two hours of stirring aqueous sodium hydroxide solution (5 ml, 2 mol/l) was added dropwise and the reaction mixture was stirred overnight. The suspension was filtered, adjusted to pH 1 with diluted hydrochloric acid and washed with ethyl acetate. The organic phase was dried in vacuo and the residue subjected to high performance liquid chromatography, yielding intermediate 4-(5-amino-1-cycloheptyl-3-oxo-1,2-dihydropyrazol-4-yl)-4-oxo-2E-butenoic acid as a pale white solid.

Yield: 95 mg (95%); ESI-MS (*m/z*): $[M+H]^+$ calc. for $C_{14}H_{20}N_3O_4^+$: 294.1 Da, found: 294.1 Da; ^{1}H-NMR (300 MHz, pyridine-d_5): δ [ppm] = 11.63 (s, NN*H*CO, 1H), 7.76 (d, $^3J_{H\text{-}H}$= 15.4 Hz, C*H*CHCOOEt, 1H), 7.52 (d, $^3J_{H\text{-}H}$ = 15.4 Hz, CHC*H*COOEt, 1H), 7.37 (s, CN*H*CO, 1H), 6.41 (s, COC*H*CNH, 1H), 4.56 (tt, $^3J_{H\text{-}H}$ = 9.4 Hz, 4.8 Hz, CC*H*$(CH_2)_2$, 1H), 2.32 - 2.20 (m, cycloheptyl-CH_2, 2H), 2.11 – 2.01 (m, cycloheptyl-CH_2, 2H), 1.73 - 1.64 (m, cycloheptyl-CH_2, 2H); 1.44 -1.41 (m, cycloheptyl-CH_2, 4H), 1.36 - 1.26 (m, cycloheptyl-CH_2, 2H). ^{13}C-NMR (75 MHz, pyridine-d_5): δ [ppm] = 168.4, 163.6, 161.8 (2), 136.4, 133.9, 86.2, 58.4, 35.5 (2), 29.1 (2), 25.1 (2).

4-(5-amino-1-cycloheptyl-3-oxo-1,2-dihydropyrazol-4-yl)-4-oxo-2E-butenoic acid (20.8 mg, 70 µmol, 1 eq) was dissolved in 20 ml ethanol. Catalytic amounts of *N,N*-dimethylformamide were added and oxalylchloride (7.2 µl, 84 µmol, 1.2 eq) were added dropwise with vigorous stirring. The reaction mixture was stirred overnight after which the solvents were evaporated. The residue was purified with reversed phase-high performance liquid chromatography, furnishing **9** as a colorless solid.

Yield: 15 mg (65%), ESI-MS (*m/z*): $[M+H]^+$ calc. for $C_{16}H_{24}N_3O_4^+$: 322.1 Da; found: 322.1 Da; ^{1}H-NMR (300 MHz, pyridine-d_5): δ [ppm] = 11.62 (s, NN*H*CO, 1H), 7.58 (d, $^3J_{H\text{-}H}$ = 15.4 Hz, NHCOC*H*CHCOOEt, 1H), 7.25 (d, $^3J_{H\text{-}H}$ = 15,4 Hz, NHCOCHC*H*COOEt, 1H), 7.15 (s, CN*H*CO, 1H), 6.44 (s, COC*H*CNH, 1H), 4.55 (tt, $^3J_{H\text{-}H}$ = 9.4 Hz, 4.8 Hz, CC*H*$(CH_2)_2$, 1H), 4.13 (q, $^3J_{H\text{-}H}$ = 7.1 Hz, $COOCH_2CH_3$, 2H), 2.35 – 2.19 (m, cycloheptyl-CH_2, 2H), 2.13 – 1.99 (m, cycloheptyl-CH_2, 2H), 1.77 – 1.60 (m, cycloheptyl-CH_2, 2H), 1.47 – 1.37 (m, cycloheptyl-CH_2, 4H), 1.39 – 1.20 (m, cycloheptyl-CH_2, 2H), 1.10 (t, $^3J_{H\text{-}H}$ = 7.1 Hz, $COOCH_2CH_3$, 3H); ^{13}C-NMR (75 MHz, pyridine-d_5): δ [ppm] = 165.8, 162.8, 161.9, 137.1, 131.7, 86.1, 61.5, 58.4, 35.6 (2), 29.0 (2), 25.1 (2), 14.4; mp.: > 225°C (decomp.)

O-Ethyl 4-(5-amino-1-phenyl-1,2-dihydropyrazol-3-one-4-yl)-4-oxo-2E-butenoate 10

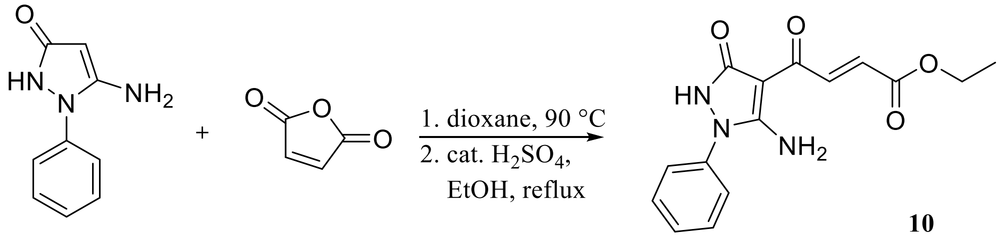

10

5-Amino-1-phenyl-1.2-dihydropyrazol-3-one (175 mg, 1 mmol, 1 eq) was dissolved in 7 ml 1,4-dioxane and transferred to a microwave vessel (pressure stable, max. volume 10 ml). Maleic anhydride (147 mg, 1.5 mmol, 1.5 eq) was added, the vessel was sealed with a septum and placed in an Initiator$^+$ microwave reactor from Biotage (Uppsala, Sweden). The solution was irradiated at 2.45 GHz with 400 W and the temperature kept at 90°C for 20 minutes.

Afterwards the solution was cooled and the solvent removed in vacuo. The residue was taken up in ethanol (15 ml) and a catalytic amount of concentrated sulfuric acid was added dropwise. After refluxing the solution for twelve hours, the solvent was evaporated and the residue subjected to reversed phase high performance liquid chromatography, which furnished **10** as an off-white solid.

Yield: 13 mg (10%); ESI-HRMS (*m/z*): [M+H]+ calc. for $C_{15}H_{16}N_3O_4^+$, 302.1135 Da; found, 302.1139 Da; ^{1}H-NMR (300 MHz, DMSO-d_6): δ [ppm] = 10.9 (s, NN*H*CO, 1H), 8.15 (d, $^3J_{H-H}$ = 15.6 Hz, C*H*CHCOOEt, 1H), 7.61 (s, NH_2, 2H), 7.56 – 7.43 (m, phenyl*H*, 3H), 7.46 – 7.35 (m, phenyl*H*, 2H), 6.65 (d, $^3J_{H-H}$ = 15.8 Hz, CHC*H*COOEt, 1H), 4.21 (q, $^3J_{H-H}$ = 7.1 Hz, COOCH_2CH$_3$, 2H), 1.26 (t, $^3J_{H-H}$ = 7.1 Hz, COOCH$_2$CH_3, 3H); ^{13}C-NMR (175 MHz, MeCN-d_3): δ [ppm] = 180.1, 165.5, 139.3, 129.6 (2), 129.4, 127.6, 127.4, 123.5 (2), 93.7, 60.7, 14.1
2 carbonyle signals could not be resolved; mp.: > 220°C (decomp.)

***O-(*5-Amino-1-cycloheptyl-1*H*-pyrazol-3-yl)-O′-ethyl but-2E-enedioate 11**

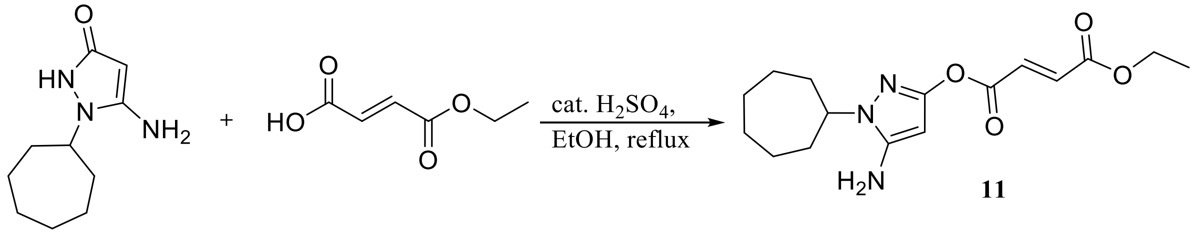

11

2 (66 mg, 0.34 mmol, 1 eq) was dissolved in pyridine (3 ml) and *N*,*N*'-dicyclohexylcarbodiimide (68 mg, 0.34 mmol, 1 eq) and mono-ethyl fumarate (47 mg, 0.34 mmol, 1 eq) were added subsequently. The suspension was stirred overnight, filtered and dried in vacuo. Purification of the residue with reversed phase high performance liquid chromatography furnished **11** as a yellow solid.

Yield: 48 mg (43%); ESI-MS (*m/z*): $[M+H]^+$ calc. for $C_{16}H_{24}N_3O_4^+$, 322.1 Da; found, 322.1 Da; ^{1}H-NMR (300 MHz, pyridine-d_5): δ [ppm] = 7.13 (d, $^3J_{H-H}$ = 15.6 Hz, OCOC*H*CHCOOEt, 1H), 7.13 (d, $^3J_{H-H}$ = 15.8 Hz, OCOCHC*H*COOEt, 1H), 5.88 (s,

NNCC*H*CNH2, 1H), 5.87 (s, NH_2, 2H), 4.54 (tt, $^3J_{H\text{-}H}$ = 9.4 Hz, 4.8 Hz, NC*H*(CH_2)$_2$, 1H), 4.17 (q, $^3J_{H\text{-}H}$ = 7.1 Hz, COOC$H_2$$CH_3$, 2H), 2.30 – 2.15 (m, cycloheptyl-CH_2, 2H), 2.14 – 2.02 (m, cycloheptyl-CH_2, 2H), 1.77 – 1.64 (m, cycloheptyl-CH_2, 2H), 1.50 – 1.41 (m, cycloheptyl-CH_2, 4H), 1.41 – 1.29 (m, cycloheptyl-CH_2, 2H), 1.15 (t, $^3J_{H\text{-}H}$ = 7.1 Hz, COOCH_2CH_3, 3H); ^{13}C-NMR (75 MHz, pyridine-d_5): δ [ppm]= 165.0, 163.1, 154.2, 147.1, 135.5, 133.1, 80.6, 61.8, 57.7, 35.0 (2), 28.8 (2), 25.0 (2), 14.4; mp.: 128-130°C

O-Ethyl N-(1-cycloheptyl-1,2-dihydropyrazol-3-one-5-yl)-3-carbamoyl-2Z-propenoate 12

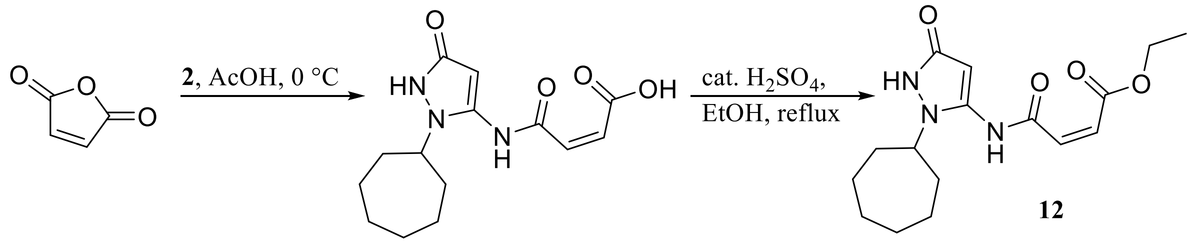

Compound **2** (120 mg (0.61 mmol, 1 eq) was dissolved in acetic acid (5 ml) cooled in an ice bath. Maleic anhydride (120 mg, 1.22 mmol, 2 eq) was added and the solution was stirred overnight. The solvent was removed in vacuo and the residue purified with flash chromatography (dichloromethane:methanol, 95:5) which yielded 4-((1-cycloheptyl-1,2-dihydropyrazol-3-one-5-yl)-amino)-4-oxo-2Z-butenoic acid as an off-white solid.

Yield: 48 mg (26%); ESI-MS (*m/z*): $[M+H]^+$ calc. for $C_{14}H_{20}N_3O_4^+$, 294.1 Da; found, 294.1 Da; TLC (dichloromethane:methanol, 4:1): R_f = 0.2; ^{1}H-NMR (300 MHz, pyridine-d_5): δ [ppm] = 13.66 (bs, NN*H*COCH, 1H), 7.68 (bs, NCN*H*, 1H), 6.63 (d, $^3J_{H\text{-}H}$ = 13.1 Hz, NHCOC*H*CHCOOH, 1H), 6.58 (s, COC*H*CNH, 1H), 6.50 (d, $^3J_{H\text{-}H}$ = 13.1 Hz, NHCOCHC*H*COOH, 1H), 4.75 (tt, $^3J_{H\text{-}H}$ = 9.3 Hz, 4.8 Hz, NC*H*(CH_2)$_2$, 1H), 2.37 – 2.20 (m, cycloheptyl-CH_2, 5H), 1.78 – 1.63 (m, cycloheptyl-CH_2, 2H), 1.53 – 1.39 (m, cycloheptyl-CH_2, 5H); ^{13}C-NMR (75 MHz, pyridine-d_5): δ [ppm] = 161.8 (3), 149.5, 136.9, 135.2, 67.5, 58.1, 35.4 (2), 29.1 (2), 25.2 (2); mp: > 200°C (decomp.)

The intermediate carboxylic acid (100 mg, 0.34 mmol) was dissolved in ethanol (5 ml) and mixed with two drops of concentrated sulfuric acid. After heating to 90°C for 60 minutes the solvent was removed in vacuo and the residue purified with reversed phase high performance liquid chromatography, which furnished **12** as an off-white solid.

Yield: 95 mg (85%); ESI-HRMS (*m/z*): $[M+H]^+$ calc. for $C_{16}H_{24}N_3O_4^+$, 322.1761 Da; found, 322.1668 Da; ^{1}H-NMR (300 MHz, pyridine-d_5): δ [ppm] = 11.54 (s, NN*H*CO, 1H), 8.54 (s, CN*H*CO, 1H), 6.83 (d, $^3J_{H\text{-}H}$ = 12.1 Hz, NHCOCHC*H*COOEt, 1H), 6.38 (d, $^3J_{H\text{-}H}$ = 12.1 Hz, NHCOC*H*CHCOOEt, 1H), 6.33 (s, COC*H*CNH, 1H), 4.23 (q, $^3J_{H\text{-}H}$ = 7.2 Hz, COOCH_2CH$_3$, 2H), 4.17 – 4.04 (m, NC*H*(CH$_2$)$_2$, 1H), 2.43 – 1.96 (m, cycloheptyl-CH_2, 4H), 1.81 – 1.26 (m, cycloheptyl-CH_2, 8H), 1.16 (t, $^3J_{H\text{-}H}$ = 7.1 Hz, COOCH$_2$CH_3, 3H); ^{13}C-NMR (75 MHz, pyridine-d_5): δ [ppm] = 166.6, 164.2, 161.8, 135.6, 135.3, 128.3, 85.9, 61.5, 58.1, 35.6 (2), 28.9 (2), 25.1 (2), 14.4; mp: 148-149°C.

O-Ethyl 3-carbamoyl-2Z-propenoate 13

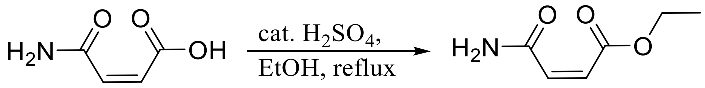

13

Maleamic acid (1.61 g, 14 mmol, 1 eq) was djssolved in EtOH. Concentrated sulfuric acid (0.15 ml, 0.2 eq) was added and the mixture was refluxed for 2.5 h. After removal of the solvent the white precipitate was purified over silica by MPLC with ethyl acetate/n-hexane 1:1, isocratic yielding **13**.

Yield:1.4 g (57%); ESI-HRMS (m/z): $[M+H]^+$ calc. for $C_6H_{10}NO_3^+$: 144.06552 Da; found: 144.06544 Da; $[M+Na]^+$, $[M+Na]^+$ calc. for $C_6H_9NO_3Na^+$: 166,04746 Da; found: 166.04785 Da; ^{1}H NMR (500 MHz, DMSO-d_6) δ [ppm] = 7.2-7.6 (m, NH_2, 2H), 6.28 (d, $^3J_{H\text{-}H}$ = 11.9 Hz, C$_2$H$_5$OCOCHC*H*CONH$_2$, 1H), 6.19 (d, $^3J_{H\text{-}H}$ = 11.9 Hz, C$_2$H$_5$OCOC*H*CHCONH$_2$, 1H),

4.10 (q, $^3J_{H\text{-}H}$ = 7.1 Hz, CH_2, 2H), 1.20 (t, $^3J_{H\text{-}H}$ = 7.1 Hz, CH_3, 3H); ^{13}C NMR (125 MHz, DMSO-d_6) δ [ppm] = 166.12, 165.82, 132.86, 127.53, 60.08, 13.88; mp: 94-95°C

N-Ethyl, N′-(1-cycloheptyl-1,2-dihydropyrazol-3-one-5-yl)-2Z-butene-diamide 14

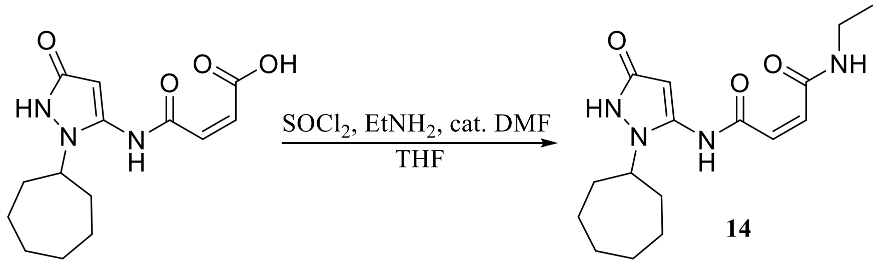

4-((1-cycloheptyl-1,2-dihydropyrazol-3-one-5-yl)-amino)-4-oxo-2Z-butenoic acid (50 mg, 0.17 mmol, 1 eq; see synthesis of **12**) was dissolved under argon in THF (5 ml). After the addition of thionyl chloride (18 µl, 2.5 mmol, 1.4 eq) and DMF (100 µl) the solution was stirred for 10 minutes. An aqueous ethylamine solution (1 ml, 30 wt.%) was added and the solution stirred for an additional two hours. The solvent was removed in vacuo and the residue subjected to reversed phase high performance liquid chromatography, which yielded product **14** as a slightly yellow solid.

Yield: 15 mg (28%); ESI-HRMS (*m/z*): $[M+Na]^+$ calc.for $C_{16}H_{24}N_4NaO_3^+$, 343.1741 Da; found, 343.1743 Da; ^{1}H-NMR (300 MHz, pyridine-d_5): δ [ppm] = 13.93 (bs, NN*H*COCH, 1H), 9.97 (bs,CN*H*CO, 1H), 7.85 (bs, CON*H*Et, 1H), 6.76 (s, COC*H*CN, 1H), 6.51 (d, $^3J_{H\text{-}H}$ = 13.4 Hz, COC*H*CHCONHEt, 1H), 6.46 (d, $^3J_{H\text{-}H}$ = 13.4 Hz, COCHC*H*CONHEt, 1H), 4.84 (tt, $^3J_{H\text{-}H}$ = 9.3 Hz, 4.5 Hz, CC*H*(CH_2), 1H), 3.43 (qd, $^3J_{H\text{-}H}$ = 7.3 Hz, 5.4 Hz, CONHC$H_2$$CH_3$, 2H), 2.41 – 2.18 (m, cycloheptyl-CH_2, 4H), 1.90 – 1.74 (m, cycloheptyl-CH_2, 2H), 1.75 – 1.59 (m, cycloheptyl-CH_2, 2H), 1.59 – 1.36 (m, cycloheptyl-CH_2, 4H), 1.15 (t, $^3J_{H\text{-}H}$ =7.3 Hz, CONHCH_2CH_3, 3H); ^{13}C-NMR (75 MHz, pyridine-d_5): δ [ppm] = 166.4, 165.6, 161.9, 161.8, 136.9, 130.4, 83.9, 58.1, 35.6 (2), 35.4, 28.8 (2), 25.5 (2), 14.5; mp: 118-120°C

***N,N'*-Diethylmaleic diamide 15**

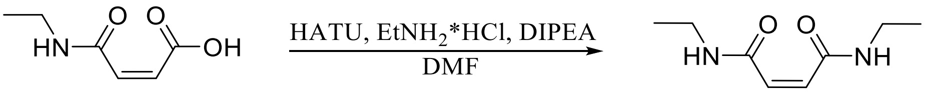

N-Ethylmaleic acid (421 mg, 2.5 mmol, 1 eq) and *N*,*N*-diisopropylethylamine (1.7 ml, 10 mmol, 4 eq) were mixed in *N*,*N*-dimethylformamide (10 ml). Ethylamine hydrochloride (205 mg, 2.5 mmol, 1 eq) and HATU coupling reagent (1.425 g, 3.75 mmol, 1.5 eq) were added and the solution was stirred for four hours. Afterwards the solution was diluted with ethyl acetate and washed six times with 5% aqueous potassium carbonate solution. The organic phase was dried with magnesium sulfate, filtered and evaporated to dryness. Normal phase flash chromatography (dichloromethane:methanol, 95:5 *v*/*v*) of the residue furnished *N*,*N*'-diethylmaleic diamide **15** as a colorless liquid.

Yield: 110 mg (25%); ^{1}H-NMR (400 MHz, DMSO-d_6): δ [ppm] = 9.18 (s, N*H*, 2H), 6.08 (s, C*H*CONH, 2H), 3.13 (q, $^{3}J_{H\text{-}H}$ = 7.3 Hz, NHCH_2CH$_3$, 4H), 1.05 (t, $^{3}J_{H\text{-}H}$ = 7.3 Hz, NHCH$_2$CH_3, 6H); ^{13}C-NMR (100 MHz, DMSO-d_6): δ [ppm] = 164.4 (2), 131.8 (2), 33.6 (2), 14.4 (2).

N-(1-Cycloheptyl-1,2-dihydropyrazol-3-one-5-yl)-prop-2-enamide 16

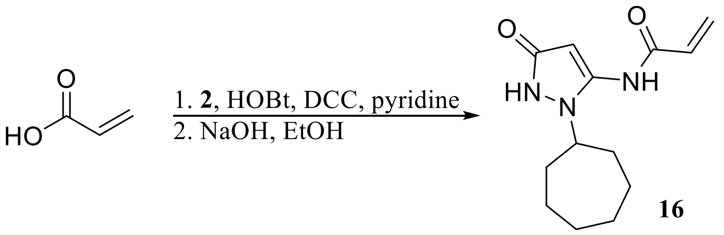

Compound **2** (100 mg, 0.5 mmol, 1 eq) and benzotriazol-1-ol (150 mg, 1.1 mmol, 2.2 eq) were dissolved in pyridine (10 ml) and *N*,*N*'-dicyclohexylcarbodiimide (226 mg, 1.1 mmol, 2.2 eq) was added. Acrylic acid (75 µl, 1.1 mmol, 2.2 eq) was added dropwise and the suspension was stirred overnight. The reaction mixture was poured into cold hexane and the precipitation was filtered, dried and dissolved in ethanol. After addition of aqueous sodium hydroxide solution (1 ml, 1 mol/l) the mixture was stirred for three hours. The solvents were

removed in vacuo and the residue purified with normal phase flash chromatography using a gradient of methylene chloride-methanol (100:0 → 90:10) as eluent. This furnished **16** as a colorless solid.

Yield: 25 mg (25%); ESI-HRMS (*m/z*): $[M+H]^+$ calc. for $C_{13}H_{20}N_3O_2^+$: 250.1550 Da, found: 250.1555 Da; ^{1}H-NMR (500 MHz, DMSO-d_6): δ [ppm] = 10.45 (s, NN*H*CO, 1H), 9.68 (s, NCN*H*CO, 1H), 7.88 (dd, $^3J_{H-H}$ = 24.9, 8.6 Hz, NHCOC*H*CH$_2$, 1H), 5.59 (d, $^3J_{H-H}$ = 8.1 Hz, NHCOCHC*H*$_2$, 1H), 5.28 (s, NNHCOC*H*, 1H), 4.74 (t, $^3J_{H-H}$ = 6.3 Hz, NHCOCHC*H*$_2$, 1H), 4.17 (tt, $^3J_{H-H}$ = 9.3, 4.9 Hz, C*H*(CH$_2$)$_2$, 1H), 1.81 – 1.70 (m, cycloheptyl-C*H*$_2$, 4H), 1.72 – 1.59 (m, cycloheptyl-C*H*$_2$, 2H), 1.61 – 1.55 (m, cycloheptyl-C*H*$_2$, 4H), 1.54 – 1.37 (m, cycloheptyl-C*H*$_2$, 2H); mp.: 230-231°C

Ethyl N-(1-(4-heptyl)-1,2-dihydropyrazol-3-one-5-yl)-carbamoyl-2Z-propenoate 17

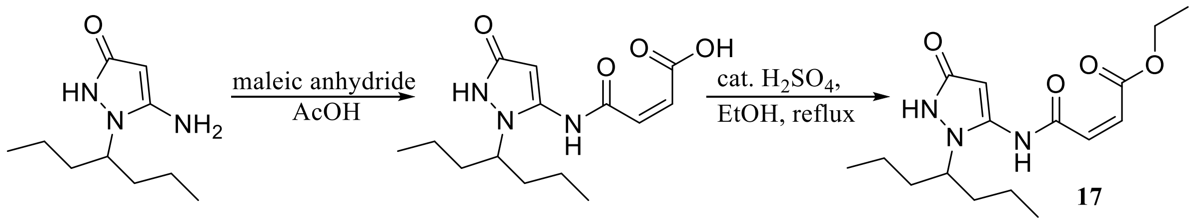

17

According to the synthetic procedure for **2**, 5-Amino-1-(4-heptyl)-1,2-dihydropyrazol-3-one was prepared by the reaction of 4-heptanone and 2-cyanoacetyl hydrazide, which yielded the desired pyrazolone after reduction with sodium borohydride.

Yield: 1.0 g (80%); ESI-MS (*m/z*): $[M-H]^+$ calc. for $C_{10}H_{18}N_3O^+$, 196.1 Da; found, 196.1 Da; ^{1}H-NMR (500 MHz, DMSO-d_6): δ [ppm] = 9.54 (s, -N*H*, 1H), 5.29 (s, -N*H*$_2$, 2H), 4.37 (s, COC*H*, 1H), 3.82 (tt, NC*H*(CH$_2$)$_2$, 1H), 1.65-1.58 (m, C*H*$_2$, 2H), 1.43-1.35 (m, C*H*$_2$, 2H), 1.20-1.13 (m, C*H*$_2$, 2H), 1.13-1.03 (m, C*H*$_2$, 2H), 0.81 (t, C*H*$_3$, 6H);^{13}C-NMR (125 MHz, DMSO-d_6): δ [ppm]= 162.9, 151.1, 73.3, 54.5, 36.3 (2), 18.9 (2),

13.9 (2); mp.: > 208°C (decomp.).

17 was prepared according to the synthetic procedure for **12**, using 5-amino-1-(4-heptyl)-1,2-dihydropyrazol-3-one as starting material.

Yield: 65 mg (33%); ESI-MS (*m/z*): $[M+H]^+$ calc. for $C_{16}H_{26}N_3O_4^+$, 324.2 Da; found, 324.2 Da; ^{1}H-NMR (500 MHz, DMSO-d_6): δ [ppm] = 10.04 (s, N*H*COCHCHCOOEt, 1H), 6.55 (d, $^3J_{H\text{-}H}$ = 11.9 Hz, NHCOC*H*CHCOOEt, 1H), 6.38 (d, $^3J_{H\text{-}H}$ = 11.9 Hz, NHCOCHC*H*COOEt, 1H), 5.50 (s, NNHCOC*H*CN, 1H), 4.12 (q, $^3J_{H\text{-}H}$ = 7.2 Hz, COOCH_2CH$_3$, 2H), 4.09 (m, NC*H*(CH_2)$_2$, 1H), 3.50 (s, NN*H*CO, 1H), 1.69 (m, heptyl-CH_2, 2H), 1.57 – 1.46 (m, heptyl-CH_2, 2H), 1.19 (t, $^3J_{H\text{-}H}$ = 7.1 Hz, COOCH$_2$CH_3, 3H), 1.17 – 1.09 (m, heptyl-CH_2, 2H), 1.00 – 0.90 (m, heptyl-CH_2, 2H), 0.79 (t, $^3J_{H\text{-}H}$ = 7.4 Hz, heptyl-CH_3, 6H); ^{13}C-NMR (125 MHz, DMSO- d_6): δ [ppm] = 165.8, 162.3, 159.4, 136.0, 131.7, 129.1, 83.4, 60.3, 55.5, 36.8 (2), 18.7 (2), 13.9, 13.8 (2).

4-(5-amino-1-cycloheptyl-3-oxo-1,2-dihydropyrazol-4-yl)-4-oxo-2E-butenoic acid (20.8 mg, 70 µmol, 1 eq) was dissolved in ethanol (20 ml) and *N*,*N*-dimethylformamide (2 µl) and oxalyl chloride (7.2 µl, 84 µmol, 1.2 eq) were added under vigorous stirring. The solution was stirred overnight after which the solvents were removed in vacuo and the residue purified with high performance liquid chromatography, yielding **17** as a colorless solid.

Yield: 65 mg (33%); ESI-MS (*m/z*): $[M+H]^+$ calc. for $C_{16}H_{26}N_3O_4^+$, 324.2 Da; found, 324.2 Da; ^{1}H-NMR (500 MHz, DMSO-d_6): δ [ppm] = 10.04 (s, N*H*COCHCHCOOEt, 1H), 6.55 (d, $^3J_{H\text{-}H}$ = 11.9 Hz, NHCOC*H*CHCOOEt, 1H), 6.38 (d, $^3J_{H\text{-}H}$ = 11.9 Hz, NHCOCHC*H*COOEt, 1H), 5.50 (s, NNHCOC*H*CN, 1H), 4.12 (q, $^3J_{H\text{-}H}$ = 7.2 Hz, COOCH_2CH$_3$, 2H), 4.09 (m, NC*H*(CH_2)$_2$, 1H), 3.50 (s, NN*H*CO, 1H), 1.69 (m, heptyl-CH_2, 2H), 1.57 – 1.46 (m, heptyl-CH_2, 2H), 1.19 (t, $^3J_{H\text{-}H}$ = 7.1 Hz, COOCH$_2$CH_3, 3H), 1.17 – 1.09 (m, heptyl-CH_2, 2H), 1.00 – 0.90 (m, heptyl-CH_2, 2H), 0.79 (t, $^3J_{H\text{-}H}$ = 7.4Hz, heptyl-CH_3, 6H); ^{13}C-NMR (125 MHz, DMSO-d_6): δ [ppm] = 165.8, 162.3, 159.4, 136.0, 131.7, 129.1, 83.4, 60.3, 55.5, 36.8 (2), 18.7 (2), 13.9, 13.8 (2).

Ethyl N-(1*H*-1,2-dihydropyrazol-3-one-5-yl)-3-carbamoyl-2Z-propenoate 18

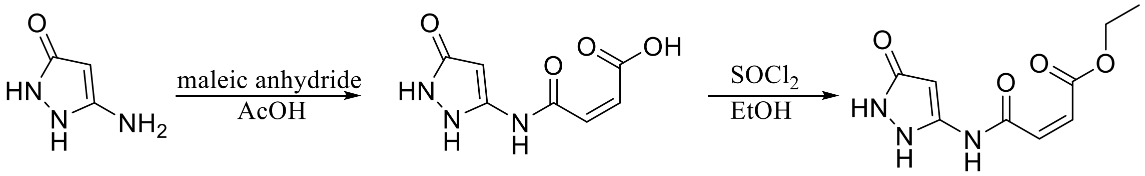

18

5-Amino-1,2-dihydro-3*H*-pyrazol-3-one[3] (350 mg, 3.55 mmol, 1 eq) was dissolved in acetic acid (30 ml) and cooled with an ice bath. Maleic anhydride (765 mg, 7.8 mmol, 2.2 eq) was added and the solution was stirred overnight. The solvent was removed in vacuo and the residue taken up in ethanol (30 ml). Thionyl chloride (386 µl, 5.3 mmol, 1.5 eq) was added dropwise and the solution was stirred overnight. Removal of the solvent in vacuo and purification of the residue via reversed phase high performance liquid chromatography furnished **18** as a colorless solid.

Yield: 80 mg (10%); ESI-MS (*m/z*): $[M+H]^+$ calc. for $C_9H_{12}N_3O_4^+$, 226.1 Da; found, 226.0 Da; ^{1}H-NMR (500 MHz, DMSO-d_6): δ [ppm] = 11.21 (s, N*H*, 1H), 10.91 (s, N*H*, 1H), 6.37 (d, $^3J_{H\text{-}H}$ = 12.0 Hz, COC*H*CHCOOEt, 1H), 6.34 (d, $^3J_{H\text{-}H}$ = 12.0 Hz, COCHC*H*COOEt, 1H), 4.91 (s, NHNHCOC*H*C, 1H), 4.02 (q, $^3J_{H\text{-}H}$ = 7.1 Hz, $COOCH_2CH_3$, 2H), 1.14 (t, $^3J_{H\text{-}H}$ = 7.1 Hz, $COOCH_2H_3$, 3H); ^{13}C-NMR (125 MHz, DMSO-d_6): δ [ppm] = 166.5, 166.1, 165.3, 158.3, 131.3, 128.9, 60.3, 33.9, 13.8; mp.: 63-64°C

N-(1-Cycloheptyl-1,2-dihydropyrazol-3-one-5-yl)-N′-(2-naphthyl)methyl-2Z-butene-diamide 19

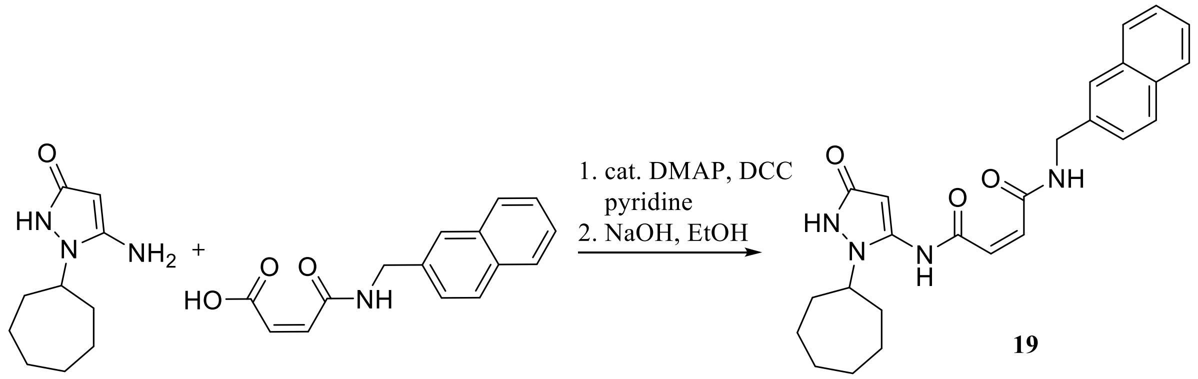

2-(Aminomethyl)naphthalene (500 mg, 3.1 mmol, 1 eq) was dissolved in methylene chloride (10 ml). Maleic anhydride (310 mg, 3.1 mmol, 1 eq) was added slowly and the solution was stirred overnight. The solvent was evaporated and the residue purified with normal phase flash chromatography using a gradient of methylene chloride and methanol (100:0 → 90:10 *v*/*v*) as eluent, which furnished 4-[(Naphthalene-2-ylmethyl)amino]-4-oxo-2Z-butenoic acid as a colorless solid.

Yield: 790 mg (98%); ESI-MS (*m/z*): $[M+H]^+$ calc. for $C_{15}H_{14}NO_3^+$, 256.0 Da; found, 256.0 Da; ^{1}H-NMR (500 MHz, DMSO-d_6): δ [ppm] = 9.53 (t, $^3J_{H-H}$ = 5.8 Hz, N*H*, 1H), 7.94 – 7.84 (m, Ar*H*, 2H), 7.83 (s, Ar*H*, 1H), 7.54 – 7.44 (m, Ar*H*, 2H), 7.43 – 7.37 (m, Ar*H*, 2H), 6.49 (d, $^3J_{H-H}$ = 12.4 Hz, COC*H*CHCO, 1H), 6.28 (d, $^3J_{H-H}$ = 12.4 Hz, COCHC*H*CO, 1H), 4.57 (d, $^3J_{H-H}$ = 5.8 Hz, CONHCH_2, 2H). The acidic proton of the carboxylic acid could not be resolved. ^{13}C-NMR (125 MHz, DMSO-d_6): δ [ppm] = 165.8, 165.3, 135.6, 132.9, 132.2, 131.7, 128.2, 128.1, 127.8, 127.6, 127.5, 126.3, 126.1, 125.9, 42.8.

2 (100 mg, 0.5 mmol, 1 eq) was dissolved in pyridine (10 ml) and 4-[(Naphthalene-2-ylmethyl)amino]-4-oxo-2Z-butenoic acid (306 mg, 1.2 mmol, 2.4 eq), 4-dimethylaminopyridine (1.2 mg, 0.01 mmol, 0.02 eq) and *N*,*N*'-dicyclohexylcarbodiimide (248 mg, 1.2 mmol, 2.4 eq) were added. The suspension was stirred overnight after which it was filtered and the solvents were coevaporated with toluene. The residue was dissolved in

ethanol (10 ml) and aqueous sodium hydroxide solution (5 mol/l, 112 µl, 0.56 mmol, 1.1 eq) was added. The solution was stirred for 30 minutes, the solvents were evaporated and the residue purified with reversed phase high performance liquid chromatography, which furnished **19** as an off-white solid.

Yield: 30 mg (13%); ESI-MS (*m/z*): $[M+H]^+$ calc. for $C_{25}H_{29}N_4O_3^+$: 433.2 Da, found: 433.0 Da; ^{1}H-NMR (500 MHz, DMSO-d_6): δ [ppm] = 10.47 (s, NN*H*CO, 1H), 8.39 (t, $^3J_{H\text{-}H}$ = 6.0 Hz, N*H*CH$_2$, 1H), 7.91 – 7.79 (m, Ar*H*, 2H), 7.69 (s, Ar*H*, 1H), 7.53 – 7.41 (m, Ar*H*, 2H), 7.46 – 7.35 (m, Ar*H*, 2H), 7.36 (s, NNHCOC*H*C, 1H), 5.55 (d, $^3J_{H\text{-}H}$ = 13.5 Hz, NHCOC*H*CHCONH, 1H), 5.54 (d, $^3J_{H\text{-}H}$ = 13.5 Hz, NHCOC*H*CHCONH, 1H), 4.48 (dd, $^3J_{H\text{-}H}$ = 15.6, 6.4 Hz, NHC*H*$_2$, 1H), 4.35 (dd, $^3J_{H\text{-}H}$ = 15.7, 5.6 Hz, NHC*H*$_2$, 1H), 4.20 (tt, $^3J_{H\text{-}H}$ = 9.1, 4.3 Hz, C*H*(CH$_2$)$_2$, 1H), 1.88 – 1.78 (m, cycloheptyl-C*H*$_2$, 4H), 1.77 – 1.62 (m, cycloheptyl-C*H*$_2$, 2H), 1.62 – 1.54 (m, cycloheptyl-C*H*$_2$, 4H), 1.55 – 1.36 (m, cycloheptyl-C*H*$_2$, 2H). One amine proton could not be resolved.

N-(1-Cycloheptyl-1,2-dihydropyrazol-3-one-5-yl)-3-(phenylsulfonyl)-2E-prop-ene-amide 20

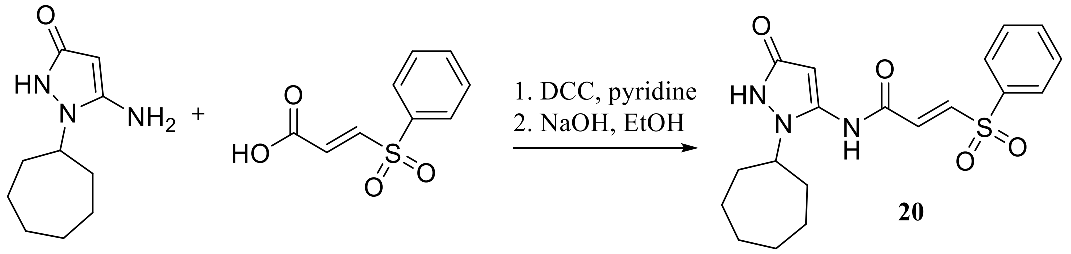

20

2 (98 mg, 0.5 mmol, 1 eq) was dissolved in pyridine (10 ml) and *N,N'*-dicyclohexylcarbodiimide (206 mg, 1 mmol, 1 eq) and (E)-3-(phenylsulfonyl)acrylic acid[4] (212 mg, 1 mmol, 1 eq) were added subsequently. The suspension was stirred overnight, filtered and dried in vacuo. Purification of the residue with reversed phase high performance liquid chromatography furnished **20** as a yellow solid.

Yield: 20 mg (10%); ESI-HRMS (*m/z*): $[M+Na]^+$ calc. for $C_{19}H_{23}N_3NaO_4S^+$, 412.1301 Da; found, 412.1292 Da; ^{1}H-NMR (500 MHz, DMSO-d_6): δ [ppm] = 10.52 (s, NCN*H*CO, 1H), 9.66 (s, NN*H*CO, 1H), 8.02 – 7.93 (m, phenyl*H*, 2H), 7.84 – 7.76 (m, phenyl*H*, 1H), 7.72 (m, phenyl*H*, 2H), 7.68 (d, $^3J_{H\text{-}H}$ = 15.0 Hz, NHCOC*H*CHSO$_2$Ph, 1H), 7.34 (d, $^3J_{H\text{-}H}$ = 15.0 Hz, NHCOCHC*H*SO$_2$Ph, 1H), 5.60 (s, NNHCOC*H*, 1H), 4.25 (tt, $^3J_{H\text{-}H}$ = 8.7, 5.5 Hz, NC*H*(CH$_2$)$_2$, 1H), 1.81 (m, cycloheptyl-C*H*$_2$, 4H), 1.77 - 1.64 (m, cycloheptyl-C*H*$_2$, 2H), 1.66 - 1.56 (m, cycloheptyl-C*H*$_2$, 2H), 1.54 – 1.38 (m, cycloheptyl-C*H*$_2$, 4H); ^{13}C-NMR (125 MHz, DMSO-d_6): δ [ppm]= 159.4, 159.3, 140.1, 138.7, 134.5 (2), 134.2 (2), 133.9, 129.9, 127.8, 83.6, 56.5, 34.3 (2), 27.9 (2), 23.9 (2); mp: > 250°C (decomp.)

N-(1-Isopropyl-1,2-dihydropyrazol-3-one-5-yl)-3-(phenylsulfonyl)-2E-prop-ene-amide 21

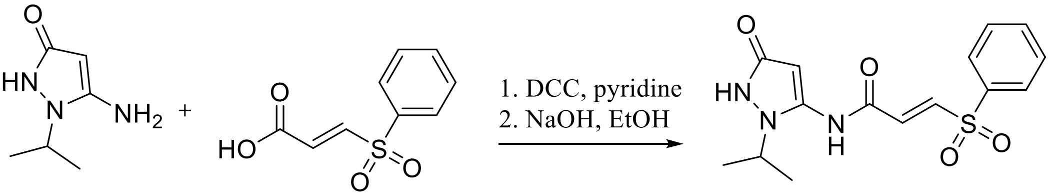

20

21 was prepared according to the synthetic procedure for **10**, using 5-Amino-1-(4-isopropyl)-1,2-dihydropyrazol-3-one as starting material.

Yield: 115 mg (35%); ESI-HRMS (*m/z*): $[M+H]^+$ calc. for $C_{15}H_{18}N_3O_4S^+$: 336.1013 Da, found: 336.1014 Da; ^{1}H-NMR (500 MHz, DMSO-d_6): δ [ppm] = 10.82 (s, NCNHCO, 1H), 10.56 (s, NN*H*CO, 1H), 7.89 (m, $J_{H\text{-}H}$ = 10.8, 8.5, 1.3 Hz, phenyl*H*, 2H), 7.84 – 7.75 (m, phenyl-*H*, 1H), 7.70 (d, $^3J_{H\text{-}H}$ = 15.1 Hz, NHCOC*H*CHSO2Ph, 1H), 7.59 – 7.52 (m, phenyl-*H*, 2H), 6.71 (d, $^3J_{H\text{-}H}$ = 15.1 Hz, NHCOCHC*H*SO2Ph, 1H), 4.58 (m, NC*H*$(CH_3)_2$, 1H), 1.22 (d, $^3J_{H\text{-}H}$ = 6.6 Hz, isopropyl-CH_3, 6H); ^{13}C-NMR (125 MHz, DMSO-d_6): δ [ppm] = 164.5, 142.2, 138.4, 134.5, 132.5, 129.8, 128.9, 127.9, 127.8, 124.4, 123.4, 85.9, 48.5, 19.68 (2); mp.: 110-112°C

Supplementary References

1. Marigo, M. Franzen, J. Poulsen, T. Zhuang, W. Jorgensen, K. Asymmetric organocatalytic epoxidation of alpha,beta-unsaturated aldehydes with hydrogen peroxide. *J. Am. Chem. Soc.* 127, 6964–6965 (2005).
2. Gyulbudaghyan, A. L. Hakopyan, M. E. Vartanyan, R. S. & Sheyranyan, M. A. A new method for the synthesis of 1-substituted 5-amino-3-hydroxypyrazoles. *Chem. J. Arm.* 55, 58–62 (2002).
3. Dickinson, R. G. & Jacobsen, N. W. The hydrazinolysis of heterocyclic compounds. II. 6-Amino-4-hydroxy-1-methylpyrimidine-2(1H)-thione and related compounds. *Aust. J. Chem.* 28, 2435 (1975).
4. Guan, Z.-H. Zuo, W. Zhao, L.-B. Ren, Z.-H. & Liang, Y.-M. An Economical and Convenient Synthesis of Vinyl Sulfones. *Synthesis* 2007, 1465–1470 (2007).
